# Multicenter International Study of the Consensus Immunoscore for the Prediction of Relapse and Survival in Early-Stage Colon Cancer

**DOI:** 10.3390/cancers15020418

**Published:** 2023-01-08

**Authors:** Bernhard Mlecnik, Alessandro Lugli, Gabriela Bindea, Florence Marliot, Carlo Bifulco, Jiun-Kae Jack Lee, Inti Zlobec, Tilman T. Rau, Martin D. Berger, Iris D. Nagtegaal, Elisa Vink-Börger, Arndt Hartmann, Carol I. Geppert, Julie Kolwelter, Susanne Merkel, Robert Grützmann, Marc Van den Eynde, Anne Jouret-Mourin, Alex Kartheuser, Daniel Léonard, Christophe Remue, Julia Wang, Prashant Bavi, Michael H. A. Roehrl, Pamela S. Ohashi, Linh T. Nguyen, SeongJun Han, Heather L. MacGregor, Sara Hafezi-Bakhtiari, Bradly G. Wouters, Giuseppe V. Masucci, Emilia K. Andersson, Eva Zavadova, Michal Vocka, Jan Spacek, Lubos Petruzelka, Bohuslav Konopasek, Pavel Dundr, Helena Skalova, Kristyna Nemejcova, Gerardo Botti, Fabiana Tatangelo, Paolo Delrio, Gennaro Ciliberto, Michele Maio, Luigi Laghi, Fabio Grizzi, Tessa Fredriksen, Bénédicte Buttard, Lucie Lafontaine, Pauline Maby, Amine Majdi, Assia Hijazi, Carine El Sissy, Amos Kirilovsky, Anne Berger, Christine Lagorce, Christopher Paustian, Carmen Ballesteros-Merino, Jeroen Dijkstra, Carlijn van de Water, Shannon van Lent-van Vliet, Nikki Knijn, Ana-Maria Mușină, Dragos-Viorel Scripcariu, Boryana Popivanova, Mingli Xu, Tomonobu Fujita, Shoichi Hazama, Nobuaki Suzuki, Hiroaki Nagano, Kiyotaka Okuno, Toshihiko Torigoe, Noriyuki Sato, Tomohisa Furuhata, Ichiro Takemasa, Prabhu Patel, Hemangini H. Vora, Birva Shah, Jayendrakumar B. Patel, Kruti N. Rajvik, Shashank J. Pandya, Shilin N. Shukla, Yili Wang, Guanjun Zhang, Yutaka Kawakami, Francesco M. Marincola, Paolo A. Ascierto, Bernard A. Fox, Franck Pagès, Jérôme Galon

**Affiliations:** 1 INSERM, Laboratory of Integrative Cancer Immunology, 75006 Paris, France; 2 Equipe Labellisée Ligue Contre le Cancer, 75006 Paris, France; 3 Centre de Recherche des Cordeliers, Sorbonne Université, Université de Paris, 75006 Paris, France; 4 Inovarion, 75005 Paris, France; 5 Institute of Pathology, University of Bern, 3008 Bern, Switzerland; 6 Immunomonitoring Platform, Laboratory of Immunology, AP-HP, Assistance Publique-Hopitaux de Paris, Georges Pompidou European Hospital, 75015 Paris, France; 7 Department of Pathology, Providence Portland Medical Center, Portland, OR 97213, USA; 8 Department of Biostatistics, M.D. Anderson Cancer Center, University of Texas, Houston, TX 77030, USA; 9 Department of Medical Oncology, University Hospital of Bern, 3010 Bern, Switzerland; 10 Pathology Department, Radboud University, 6500 HC Nijmegen, The Netherlands; 11 Department of Pathology, University Erlangen-Nürnberg, 91054 Erlangen, Germany; 12 Department of Surgery, University Erlangen-Nürnberg, 91054 Erlangen, Germany; 13 Institut Roi Albert II, Department of Medical Oncology, Cliniques Universitaires St-Luc, 1200 Brussels, Belgium; 14 Institut de Recherche Clinique et Experimentale (Pole MIRO), Université Catholique de Louvain, 1200 Brussels, Belgium; 15 Department of Pathology, Cliniques Universitaires St-Luc, 1200 Brussels, Belgium; 16 Institut de Recherche Clinique et Experimentale (Pole GAEN), Université Catholique de Louvain, 1200 Brussels, Belgium; 17 Institut Roi Albert II, Department of Digestive Surgery, Cliniques Universitaires St-Luc Université Catholique de Louvain, 1200 Brussels, Belgium; 18 Curandis, New York, NY 10583, USA; 19 Department of Pathology, Laboratory Medicine Program, University Health Network, 11-E444, Toronto, ON M5G 2C4, Canada; 20 Department of Laboratory Medicine and Pathobiology, University of Toronto, Toronto, ON M5S 1A8, Canada; 21 Department of Pathology, Memorial Sloan Kettering Cancer Center, New York, NY 10065, USA; 22 Princess Margaret Cancer Centre, Toronto, ON M5G 2C1, Canada; 23 Department of Oncology-Pathology, Karolinska Institutet, Karolinska University, 17177 Stockholm, Sweden; 24 Department of Oncology, First Faculty of Medicine, General University Hospital in Prague, Charles University, 12808 Prague, Czech Republic; 25 Institute of Pathology, First Faculty of Medicine, General University Hospital in Prague, Charles University, 12808 Prague, Czech Republic; 26 Department of Pathology, Istituto Nazionale Tumori IRCCS Fondazione G. Pascale, 80131 Napoli, Italy; 27 Colorectal Surgery Department, Instituto Nazionale Tumori IRCCS Fondazione G. Pascale, 80131 Napoli, Italy; 28 IRCCS Istituto Nazionale Tumori “Regina Elena”, 00128 Rome, Italy; 29 Center for Immuno-Oncology, University Hospital, 53100 Siena, Italy; 30 Laboratory of Molecular Gastroenterology, IRCCS Humanitas Research Hospital, Rozzano, 20090 Milan, Italy; 31 Department of Medicine and Surgery, University of Parma, 43125 Parma, Italy; 32 Department of Immunology and Inflammation, IRCCS Humanitas Research Hospital, Rozzano, 20090 Milan, Italy; 33 Department of Biomedical Sciences, Humanitas University, Pieve Emanuele, 20072 Milan, Italy; 34 Digestive Surgery Department, AP-HP, Assistance Publique-Hopitaux de Paris, Georges Pompidou European Hospital, 75015 Paris, France; 35 Department of Pathology, AP-HP, Assistance Publique-Hopitaux de Paris, Georges Pompidou European Hospital, 75015 Paris, France; 36 Department of Molecular Microbiology and Immunology, Oregon Health and Science University, Portland, OR 97239, USA; 37 Department of Surgical Oncology, Regional Institute of Oncology, University of Medicine and Pharmacy “Grigore T. Popa”, 700115 Iaşi, Romania; 38 Division of Cellular Signaling, Institute for Advanced Medical Research, School of Medicine, Keio University, Tokyo 160-8582, Japan; 39 Department of Translational Research and Developmental Therapeutics against Cancer, Yamaguchi University School of Medicine, Yamaguchi 755-8505, Japan; 40 Department of Gastroenterological, Breast and Endocrine Surgery, Yamaguchi University Graduate School of Medicine, Yamaguchi 753-8511, Japan; 41 Department of Surgery, School of Medicine, Kindai University, Osaka-sayama 589-0014, Japan; 42 Department of Pathology, Sapporo Medical University, Sapporo 060-8556, Japan; 43 Department of Surgery, Surgical Oncology, and Science, Sapporo Medical University, Sapporo 060-8556, Japan; 44 The Gujarat Cancer & Research Institute, Asarwa, Ahmedabad 380016, India; 45 Institute for Cancer Research, School of Basic Medical Science, Xi’an 710061, China; 46 Health Science Center of Xi’an Jiaotong University, Xi’an 710061, China; 47 Kite Pharma, Santa Monica, CA 90404, USA; 48 Melanoma Cancer Immunotherapy and Innovative Therapies Unit, Istituto Nazionale Tumori IRCCS Fondazione “G. Pascale”, 80131 Napoli, Italy; 49 Laboratory of Molecular and Tumor Immunology, Earle A. Chiles Research Institute, Robert W. Franz Cancer Center, Providence Portland Medical Center, Portland, OR 97213, USA

**Keywords:** Immunoscore, colon cancer, prognosis, predictive biomarkers, early-stage, tumor microenvironment

## Abstract

**Simple Summary:**

This work determines the predictive value of the consensus Immunoscore in 1885 patients with AJCC/UICC-TNM stage I-II Colon Cancer (CC) from North American, European, and Asian care centers. Herein, we demonstrate that the immunity of early-stage CC patients, more than cancer cell-associated parameters, predicts outcome for stage I/II patients. Similar results were found for high-risk patients defined based on parameters such as the grade of differentiation, T4 stage, venous emboli, lymphatic invasion or perineural invasion (VELIPI). Within these pathological risk subgroups, the consensus Immunoscore accurately identifies early-stage CC patients with different clinical outcome, without treatment bias. Thus, the Immunoscore reliably diagnoses low immune cell infiltrated patients at risk of relapse, that would benefit from a more frequent and detailed medical monitoring. The Immunoscore is a patient classification method that can guide treatment decisions, through the quantification of CD3+ and cytotoxic CD8+ T-lymphocytes densities within the tumor and its invasive margin.

**Abstract:**

Background: The prognostic value of Immunoscore was evaluated in Stage II/III colon cancer (CC) patients, but it remains unclear in Stage I/II, and in early-stage subgroups at risk. An international Society for Immunotherapy of Cancer (SITC) study evaluated the pre-defined consensus Immunoscore in tumors from 1885 AJCC/UICC-TNM Stage I/II CC patients from Canada/USA (Cohort 1) and Europe/Asia (Cohort 2). METHODS: Digital-pathology is used to quantify the densities of CD3+ and CD8+ T-lymphocyte in the center of tumor (CT) and the invasive margin (IM). The time to recurrence (TTR) was the primary endpoint. Secondary endpoints were disease-free survival (DFS), overall survival (OS), prognosis in Stage I, Stage II, Stage II-high-risk, and microsatellite-stable (MSS) patients. RESULTS: High-Immunoscore presented with the lowest risk of recurrence in both cohorts. In Stage I/II, recurrence-free rates at 5 years were 78.4% (95%-CI, 74.4–82.6), 88.1% (95%-CI, 85.7–90.4), 93.4% (95%-CI, 91.1–95.8) in low, intermediate and high Immunoscore, respectively (HR (Hi vs. Lo) = 0.27 (95%-CI, 0.18–0.41); *p* < 0.0001). In Cox multivariable analysis, the association of Immunoscore to outcome was independent (TTR: HR (Hi vs. Lo) = 0.29, (95%-CI, 0.17–0.50); *p* < 0.0001) of the patient’s gender, T-stage, sidedness, and microsatellite instability-status (MSI). A significant association of Immunoscore with survival was found for Stage II, high-risk Stage II, T4N0 and MSS patients. The Immunoscore also showed significant association with TTR in Stage-I (HR (Hi vs. Lo) = 0.07 (95%-CI, 0.01–0.61); *P* = 0.016). The Immunoscore had the strongest (69.5%) contribution χ^2^ for influencing survival. Patients with a high Immunoscore had prolonged TTR in T4N0 tumors even for patients not receiving chemotherapy, and the Immunoscore remained the only significant parameter in multivariable analysis. CONCLUSION: In early CC, low Immunoscore reliably identifies patients at risk of relapse for whom a more intensive surveillance program or adjuvant treatment should be considered.

## 1. Introduction

The AJCC/UICC-TNM cancer staging system provides helpful, yet incomplete prognostic information for early-stage colon cancer [1]. Cancer classifications based on tumor cell characteristics [1,2] have only shown a moderate prediction accuracy and clinical usefulness. Risk assessment is particularly important to decide when to propose an adjuvant treatment for Stage II CC patients. High-risk Stage II patients, defined as those with commonly poor prognostic features including T4 tumors, lymph nodes sampling <12, poorly differentiated tumors, lymphatic/vascular or perineural invasion, bowel obstruction or perforation, can be considered for adjuvant chemotherapy. However, these risk features are imperfect and additional risk factors are needed to guide treatment decisions.

Similarly, in Stage I, survival rates are high and adjuvant chemotherapy is not typically recommended. However, approximately 10% of Stage I CC tumors will recur even after surgical resection [3,4,5]. Thus, the precise histologic evaluation of resected specimens is necessary for deciding further treatment strategies, including chemotherapy. Several histologic factors have been proposed for evaluating the risk of lymph node metastasis of Stage I CC, with positive lymphatic/vascular or perineural invasion, positive poorly differentiated tumor and deep (≥1000 μm) submucosal invasion, as the main risk factors of lymph-node metastasis [3,4,6]. Furthermore, the tumor budding, a major histological characteristic in colorectal carcinoma, is validated as a prognostic factor of tumor progression and included among the high-risk factors, especially in early-stages I-II colorectal carcinoma [7,8,9].

The Immunoscore is an in vitro diagnostic test that predicts the risk of relapse in patients with Colon Cancer (CC) by measuring the host immune response at the tumor site [10,11]. It is a risk-assessment tool that quantifies both CD3+ lymphocytes and CD8+ cytotoxic T cells in the CT and IM. This immune scoring system provides independent and superior prognostic value than traditional risk parameters and is intended for use as an adjunct to the TNM classification [12].

In situ tumor-infiltrating immune cells have been associated with a favorable prognostic outcome [1,12,13,14,15,16,17,18,19,20,21,22,23,24,25,26,27]. In CRC, we have shown that the strength of the in situ adaptive immune reaction at the center of the tumor (CT) and the invasive margin (IM), widely correlates with patient time to recurrence (TTR) and overall survival (OS) [12,15,20,21,25,26]. We defined the immune contexture as major determinant of clinical outcome in patients with colorectal cancer [14,15,28]. The Immunoscore was shown to predict clinical outcome in early [12,26,29,30,31] and advanced [32,33,34,35,36] stage CRC patients. Recently, an international consortium, led by the Society for Immunotherapy of Cancer, validated the consensus Immunoscore assay in patients with TNM Stage I-III CC [12]. Patients with a high Immunoscore had the lowest risk of recurrence at 5 years compared to those with a low Immunoscore. The prognostic and predictive value of the Immunoscore in response to chemotherapy were validated in Stage III CC patients [37,38,39,40,41]. However, the prognostic value of the consensus Immunoscore in predicting the risk of recurrence and death, in CC Stage I, Stage II, and high-risk Stage II tumors, had not been previously described and thus remains unclear. In this study, the international SITC Immunoscore consortium aimed at validating the pre-defined consensus Immunoscore in patients with early-stage CC. Herein, we report the Immunoscore results to stratify Stage I, Stage II, high-risk Stage II, and microsatellite-stable (MSS) CC patients, with clinical implications.

## 2. Materials and Methods

### 2.1. Patients

An international consortium composed of 14 pathology expert centers from 13 countries was initiated to evaluate the standardized Immunoscore assay in primary tumors from 1885 patients with Stage I/II CC. Patients were split into two cohorts (North America (USA + Canada) and Europe + Asia). Patients who received preoperative treatment were systemically excluded. Clinical data from North America and Europe and Asia datasets are presented in Table S1. Outcomes of interest were TTR, defined as the elapsed time from surgery until the first recurrence of disease, OS stands for the delay from surgery to death due to any cause and DFS is the overall patient survival time without any symptoms, from surgery until the first event of relapse or death. High-risk Stage II patients were defined as VELIPI+ (venous emboli or lymphatic invasion or perineural invasion) or T4 Stage II tumors, or perforation, or poor differentiation or less than 12 lymph-node (LN) evaluated, whereas low-risk correspond to T1-3 stage tumors without any high-risk feature. In each center, an ethical review board approved the ethical, legal, and social implications.

### 2.2. Immunohistochemistry

For each patient, a tumor block containing the CT and the IM is selected by the pathologist of every care center. According to the reference center protocol and as previously described [12], two tissue paraffin sections of 4 microns were processed for immunohistochemistry. Digital slides were scanned at 20× magnification with a resolution of 0.45 µm/pixel.

### 2.3. Image Analysis

A specially developed Immunoscore module (INSERM, Paris, France), integrated into the image analysis system Developer XD (Definiens, Munich, Germany has been used to determine cell densities of stained CD3+ and CD8+, in CT and IM regions. The mean and the distribution of the staining intensities were monitored, providing an internal quality control of each slide.

### 2.4. Immunoscore Determination

For each slide, CD3+ and CD8+ cell densities in CT and IM regions were converted into percentiles [12]. The mean of the four percentiles obtained (two markers, two regions) was calculated and translated into the Immunoscore scoring system. The Immunoscore categories were previously pre-defined, independently of clinical data [12]. These pre-defined categories were used. Immunoscore 3 categories, in which the mean percentiles are the following: Low (Lo) 0–25%, Intermediate (Int) (>25–70%) and High (Hi) (>70–100%). Additional analyses were performed with the pre-defined Immunoscore 2 categories: Lo (0–25%) and Int + Hi (25–100%) and Immunoscore 5 categories: I0 (0–10%), I1 (>10–25%), I2 (>25–70%), I3 (>70–95%) and I4 (>95–100%).

### 2.5. Monitoring of the Study

The biomarker reference center (Immunomonitoring platform, Hôpital Européen Georges Pompidou AP-HP, INSERM, Paris) optimized immunostaining protocols, supplied the user manual for Immunoscore software and validated data from each cohort, analyzed by the 14 participating centers [12]. Exclusion criteria are missing counts at either tumor region or low staining intensity (≤152 AU). Analyses were performed on 1885 patients with early-stage CC including 1434 Stage II and 451 Stage I CC patients.

### 2.6. MSI Status

In patients with sufficient samples available (*n* = 476), genomic DNA was extracted from paired tumor and normal colonic tissue, out of formalin-fixed paraffin-embedded (FFPE) slides. MSI status was assessed with the molecular new Bethesda panel. Patients with deficient mismatched repair and proficient mismatched repair were denoted MSI and MSS, respectively.

### 2.7. Statistics

Demographics and disease characteristics were descriptively compared across the North America and Europe and Asia. They were also compared using Student’s *t*-test and Chi-square (χ^2^) test, when applicable. Bivariable association between Immunoscore and time-to-event outcomes was analyzed by the log-rank test, stratified Cox proportional hazards model by participating center and by restricted mean survival time (RMST) (survRM2, R package), with time cutoff based on the group with the shortest follow-up [12,42,43]. To evaluate associations between Immunoscore and outcomes adjusting for potential confounders, stratified multivariable Cox models have been used. Model performance was assessed by Harrell’s C-statistics. Each center had been used as a stratification factor, and the variables adjusted in the multivariable models were gender, T-stage, N-stage and MSI. The likelihood ratio test *P*-value was used for comparing the performance of risk prediction models. χ^2^ from Harrell’s RMS R package served to assess the relative importance of each parameter to survival risk.

## 3. Results

### 3.1. Immunoscore and the Outcome of Stage I/II Colon Cancer Patients

Biomarker data from 1885 colon cancer patients with AJCC/UICC-TNM Stage I/II part of the Immunoscore international validation study were investigated (see consort diagram in Supplementary Information). Patients were divided into two datasets: cohort 1 (North America) and cohort 2 (Europe and Asia) with balanced demographic and clinical characteristics (Table S1). Overall, 52.6% of patients were males, with 68 years as a median age (IQR 60–77). The mean number of lymph-nodes examined was 19.0. Among all analyzed patients, 223 relapses (11.8% of patients) and 588 deaths (31.2% of patients) were observed. Relapses were observed for 24/451 (5, 3%) of stage I and 199/1434 (13, 9%) of stage II patients. The median follow-up time for censored patients was 69.5 months (95% CI, 67.0–71.6), and the median survival time from surgery to death due to any cause was 94.9 months (95% CI, 91.0–99.4).

**Figure 1:**
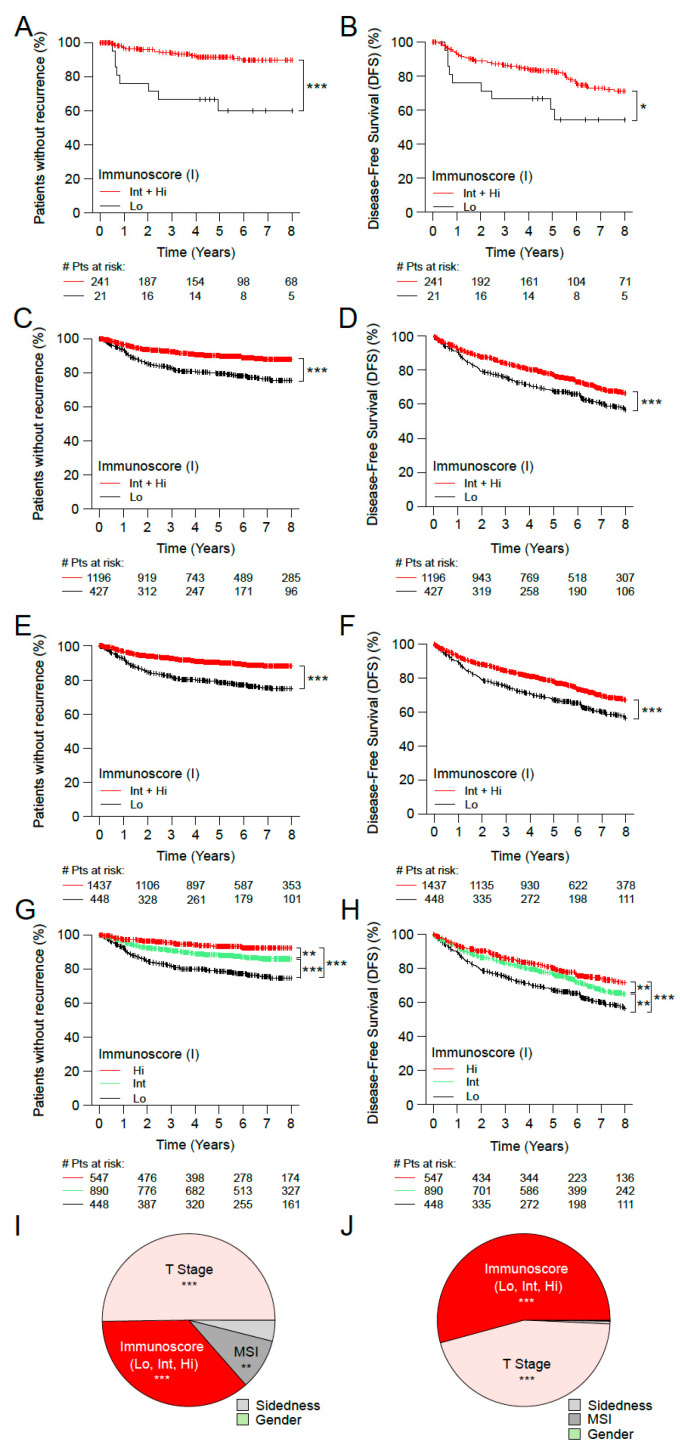
The impact of Immunoscore on the outcome of patients with early-stage (Stage I/II) colon cancer. Kaplan-Meier curves of Immunoscore (I) are shown for TTR (**A,C,E,G**) and DFS (**B,D,F,H**) for patients from cohort 1 (**A,B**), cohort 2 (**C,D**), all patients from cohort 1+2 (**E–H**). (**A–F**) Immunoscore two categories: I Lo (0–25%, black), I Int + Hi (>25–100%, red). (**G,H**) Immunoscore three categories: I Lo (0–25%, black), I Int (>25–70%, green) and I Hi (>70–100%, red). Relative importance of each risk parameter to survival risk for TTR (**I**) and DFS (**J**) using the χ^2^ proportion test for clinical parameters and Immunoscore corresponding to panels G and H. Significant logrank *p*-values are marked as *** *p* < 0.001, ** 0.001 < *p* ≤ 0.01, * 0.01 < *p* ≤ 0.05.

The densities of CD3+ and CD8+ positive T-cells were converted into the consensus Immunoscore categories using the same pre-defined cut-points, like previously reported [12]. In early-stage CC patients (*n* = 1885), the prognostic value of the Immunoscore was further evaluated in cohorts 1 and 2 (Figure 1, Figure S1). In cohort 1, the two categories of Immunoscore permitted the identification of patients with significant different clinical outcome for TTR and DFS (*n* = 262, Figure 1A,B and Table S2). Patients with Int+Hi Immunoscore had a significantly lower risk to relapse and a prolonged TTR (5 years recurrence rate Int + Hi: 8.6%, Lo: 40.0%; unadjusted HR Int + Hi vs. Lo = 0.19 95% CI, 0.08–0.43; *p* < 0.0001) and DFS (5 years survival rate Int + Hi: 83.1%, Lo: 60.6%; unadjusted HR Int + Hi vs. Lo = 0.48 95% CI, 0.24–0.97; *p* = 0.0399) (Table S2). The two category Immunoscore was validated in the cohort 2 (*n* = 1623, Figure 1C,D; Table S2) using the same methodology and cut-points. In cohort 2, patients with Int + Hi Immunoscore showed a significant reduction in their risk of relapse (5 years recurrence rate Int + Hi: 10.2%, Lo: 20.6%; unadjusted HR Int + Hi vs. Lo = 0.47 95% CI, 0.35–0.63; *p* < 0.0001) and DFS (5 years survival rate Int + Hi: 77.0%, Lo: 67.6%; unadjusted HR Int + Hi vs. Lo = 0.68 95% CI, 0.57–0.81; *p* < 0.0001) (Figure 1C,D; Table S2).

Among all patients with early-stage CC (*n* = 1885, Figure 1E,F, Table 1 and Table S3), 76.2% were classed Int + Hi (Figure 1E,F and Table 1). These patients showed a significant reduced rate of relapse (5 years recurrence rate Int + Hi: 10.0%, Lo: 21.6%; unadjusted HR Int + Hi vs. Lo = 0.43 95% CI, 0.32–0.57; *p* < 0.0001) and DFS (5 years survival rate Int + Hi: 78.0%, Lo: 67.3%; unadjusted HR Int + Hi vs. Lo = 0.67 95% CI, 0.56–0.79; *p* < 0.0001) (Table 1).

In three category Immunoscore, patients with high (29.0%), intermediate (47.2%), and low (23.8%) Immunoscore presented with recurrence rates at 5 years of 6.6%, 11.9%, and 21.6%, respectively (unadjusted HR Hi vs. Lo = 0.27; 95% CI, 0.18–0.41; *p* < 0.0001) (Figure 1G,H and Table 1).

Cox multivariable analyses for all early-stage patients assessed for Immunoscore, gender, T-stage, MSI, sidedness, and stratified by center, revealed a significant prognostic value of the Immunoscore three categories (TTR: [Hi vs. Lo, HR = 0.29 95% CI, 0.17–0.5; *p* < 0.0001], DFS: [Hi vs. Lo, HR = 0.48 95% CI, 0.35–0.65); *p* < 0.0001]) (Figure 1I,J and Table S4). Variables with major relative impact on the risk (χ^2^) were: T-stage, the Immunoscore, and MSI in TTR (Figure 1I), and in the DFS: the Immunoscore and T-stage (Figure 1J). Other parameters have only shown a minor relative contribution (<10%) (Figure 1I,J). When considered as a continuous variable, the Immunoscore remained significant without dichotomization in a multivariable analysis for TTR and DFS (all *p* < 0.001). Furthermore, introducing the Immunoscore to a model that includes all clinical variables has improved considerably the prediction for TTR (likelihood ratio test, *p* < 0.0001) and DFS (likelihood ratio test, *p* < 0.0001) (Figure S4 and Table S4).

### 3.2. Immunoscore, Time-to-Event and Survival among Microsatellite Stable (MSS) Patients with Stage II Disease

**Figure 2:**
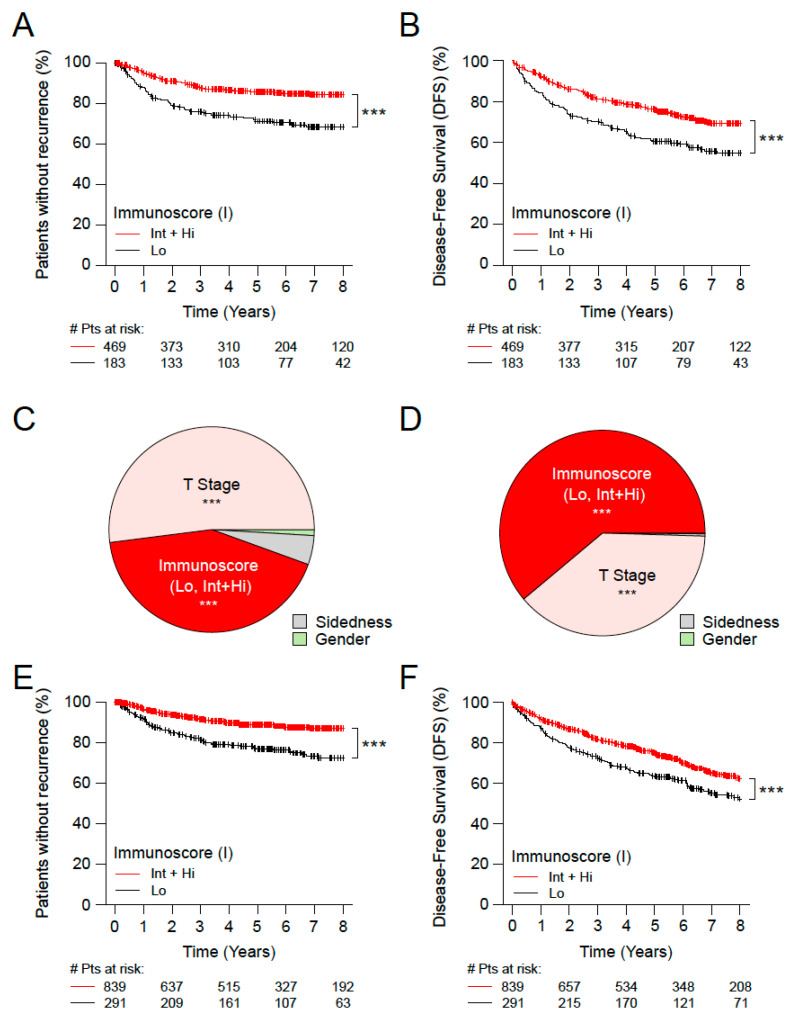
The impact of Immunoscore on MSS patients with Stage II colon cancer. Kaplan-Meier curves of Immunoscore (I) two categories: Lo (0–25%, black) and Int+Hi (>25–100%, red) are shown for TTR (**A,E**) and DFS (**B,F**). (**A–D**) Stage II MSS patients from cohorts 1 and 2. (**E,F**) Untreated Stage II T4N0 patients from cohorts 1 and 2. Relative importance of each risk parameter to survival risk for TTR (**C**) and DFS (**D**) using the χ^2^ proportion test for clinical parameters and Immunoscore corresponding to panels A, B. Significant logrank *p*-values are marked as *** *p* < 0.001.

Patients with MSS features can be considered at higher risk than MSI patients with a favorable outcome. The Immunoscore was thus investigated in the subgroup of MSS Stage II CC patients (Table 1 and Table S3). Stage II CC patients with MSS identified with Int + Hi Immunoscore presented a significantly prolonged survival for TTR (5 years recurrence rate Int + Hi: 14.1%, Lo: 28.7%; unadjusted HR [Int + Hi vs. Lo] = 0.49; 95% CI, 0.36–0.66; *p* < 0.0001) and DFS (5 years recurrence rate Int + Hi: 75.8%, Lo: 60.6%; unadjusted HR [Int + Hi vs. Lo] = 0.71; 95% CI, 0.58–0.85; *p* = 0.0003) compared to low Immunoscore patients (Figure 2A,B, Table 1 and Table S3). The Immunoscore and T-stage had the most important relative contribution to the risk in TTR and DFS while Gender and sidedness had a small relative contribution (Figure 2C,D). Similar results were obtained for the OS (Figure S2A,B). The Immunoscore in three categories further increased the differences in TTR and DFS between Immunoscore low and high (Table 1 and Table S3).

In tumors from all Stage II patients who did not receive chemotherapy (*n* = 1130), the Immunoscore [Int + Hi vs. Lo] was significantly (*p* < 0.0001) associated with TTR and DFS (Figure 2E,F and Table S4). This finding was also valid in the subgroup of MSS patients (Figure S2E,F and Table S3). Similar significant results were found for three category Immunoscore (*p* < 0.001) in untreated patients (Figure S2C,D and Table S4).

### 3.3. Immunoscore, Time-to-Event and Survival among Patients with High-Risk and Low-Risk Stage II Disease

Stage II patients with particular high risk are the ones with expended primary tumor (T4), VELIPI+, poor histologic differentiation, bowel perforation or less than 12 sampled peripheral lymph nodes. A patient having at least one of these tumor related parameters was considered at high risk. A very high-risk category was defined based on T4 primary tumors and VELIPI+, as well as an additional group with T4N0 tumors only.

**Figure 3:**
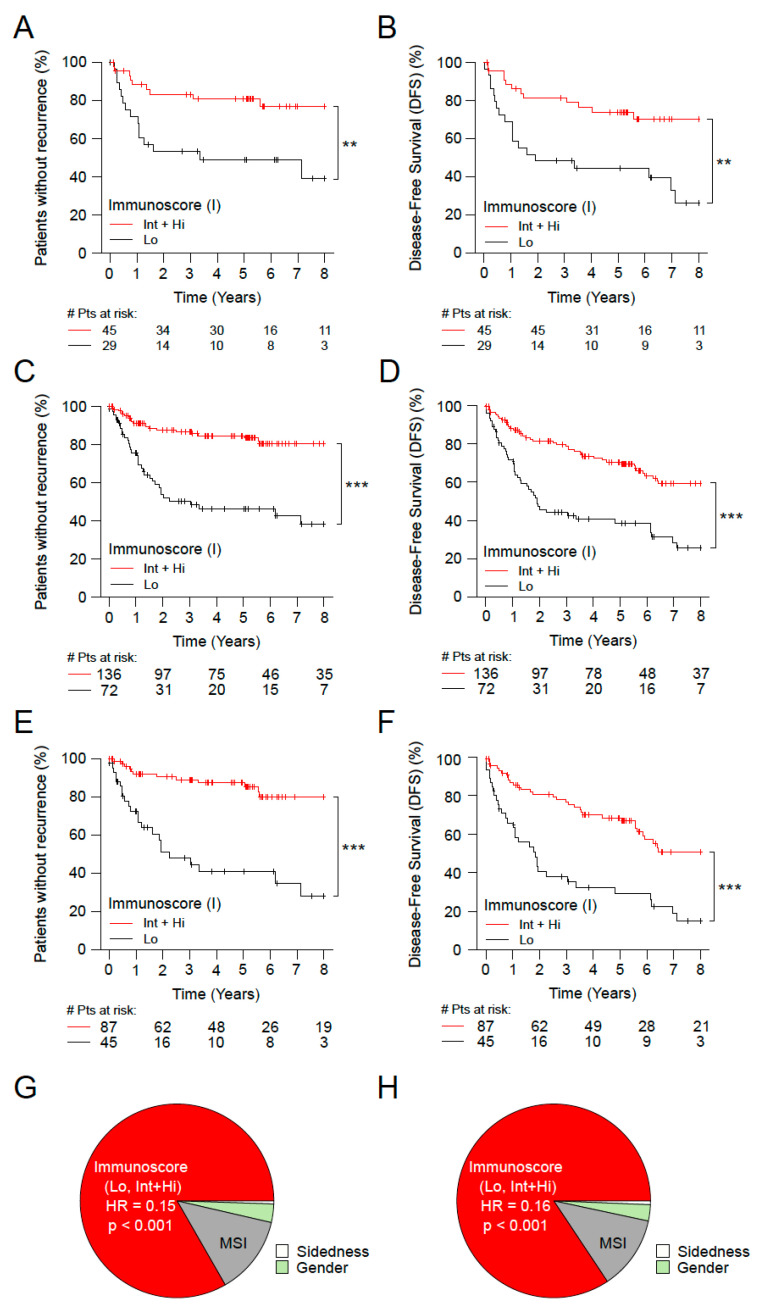
The impact of Immunoscore on very high-risk patients with Stage II colon cancer. Kaplan-Meier curves of Immunoscore (I) two categories: Lo (0–25%, black) and Int+Hi (>25–100%, red) are shown for TTR (**A,C,E**) and DFS (**B,D,F**) for subgroups of Stage II patients from cohorts 1 and 2 with very high risk (T4 primary tumors and VELIPI+) (**A,B**), T4N0 patients (**C,D**), and T4N0 patients who did not received chemotherapy (**E,F**). Relative importance of each risk parameter to survival risk for TTR (**G**) and DFS (**H**) using the χ^2^ proportion test for clinical parameters and Immunoscore corresponding to panels C and D. Significant logrank *p*-values are marked as *** *p* < 0.001, ** 0.001 < *p* ≤ 0.01.

Among all Stage II patients (*n* = 1434), these three patient risk groups were investigated in relation to consensus Immunoscore. In all risk groups, high risk, very high (Figure 3A,B and Figure S2G–J) and T4N0 (Figure 3C,D) patients that had a high Immunoscore were also the ones to have a prolonged survival (Table S3).

The Immunoscore assessment of Stage II patients with very high-risk showed a significant association with TTR (5 years survival rate Hi: 15.6%, Int: 20.9%, Lo: 51.1%; unadjusted HR [Hi vs. Lo] = 0.23; (95% CI, 0.04–1.37); *p* = 0.1061) and DFS (5 years survival rate Hi: 69.6%, Int: 76.4%, Lo: 44.3%; unadjusted HR [Hi vs. Lo] = 0.36; (95% CI, 0.09–1.47); *p* = 0.1560) (Figure 3A,B and Table S3). In three category Immunoscore (*p* < 0.0005), similar significant results were also found (Figure S2G–J). Strikingly, patients with high-risk or very high-risk Stage II, classified with Int + Hi Immunoscore present a good outcome, similar to the rest of Stage II cohort, with lower risk (Figure S3A–D and Table S4). Within the risk group, patients with high Immunoscore had a significantly prolonged survival compared to low infiltrated tumors, independently if they were treated or not with chemotherapy (Figure S3E).

The proportion of Immunoscore within the T4N0 population was 65.4% with Int + Hi Immunoscore and 34.6% with low Immunoscore (cohorts 1+2, Table S3). The Immunoscore was highly and significantly associated with TTR and DFS within the subgroup of T4N0 tumors (*p* < 0.0001) (Figure 3C,D and Table S3). The 5 years DFS rates were 70.5% for Int+Hi Immunoscore, and 38.5% for low Immunoscore (unadjusted HR [Int + Hi vs. Lo] = 0.31 (95% CI 0.19–0.49), *p* < 0.0001). Furthermore, similar results were found in the population of T4N0 who did not received chemotherapy, with the Immunoscore significantly associated with TTR (unadjusted HR [Int + Hi vs. Lo] = 0.12 (95% CI 0.05–0.28), *p* < 0.0001) and with DFS (unadjusted HR [Int + Hi vs. Lo] = 0.25 (95% CI 0.15–0.44), *p* < 0.0001) (Figure 3E and Table S3). Similar results were found using restricted mean survival time without recurrence (RMST) with a difference of 80.9 months between high Immunoscore and low Immunoscore (*p* < 0.0001). The relative importance of each risk parameter to survival risk for TTR and DFS, using the χ^2^ proportion test for clinical parameters and Immunoscore, was investigated. This revealed that the Immunoscore has the highest contribution to predict TTR and DFS (>76%), making it stronger than all the other parameters (*p* < 0.001) (Figure 3G,H). Cox Multivariable analysis in Stage II T4N0 colon cancer patients, showed that the Immunoscore was the only remaining significant parameter (TTR: [Hi vs. Lo], HR = 0.15 (95% CI, 0.05–0.46), *p* = 0.0009). In contrast, gender, sidedness, mucinous, grade of differentiation, VELIPI, MSI were not significant (Table S4). Thus, the Immunoscore significantly predicted survival in early-stage CC, in Stage II and in sub-groups of high-risk Stage II CC.

### 3.4. Immunoscore and the Outcome of Stage I MSS Colon Cancer Patients

**Figure 4:**
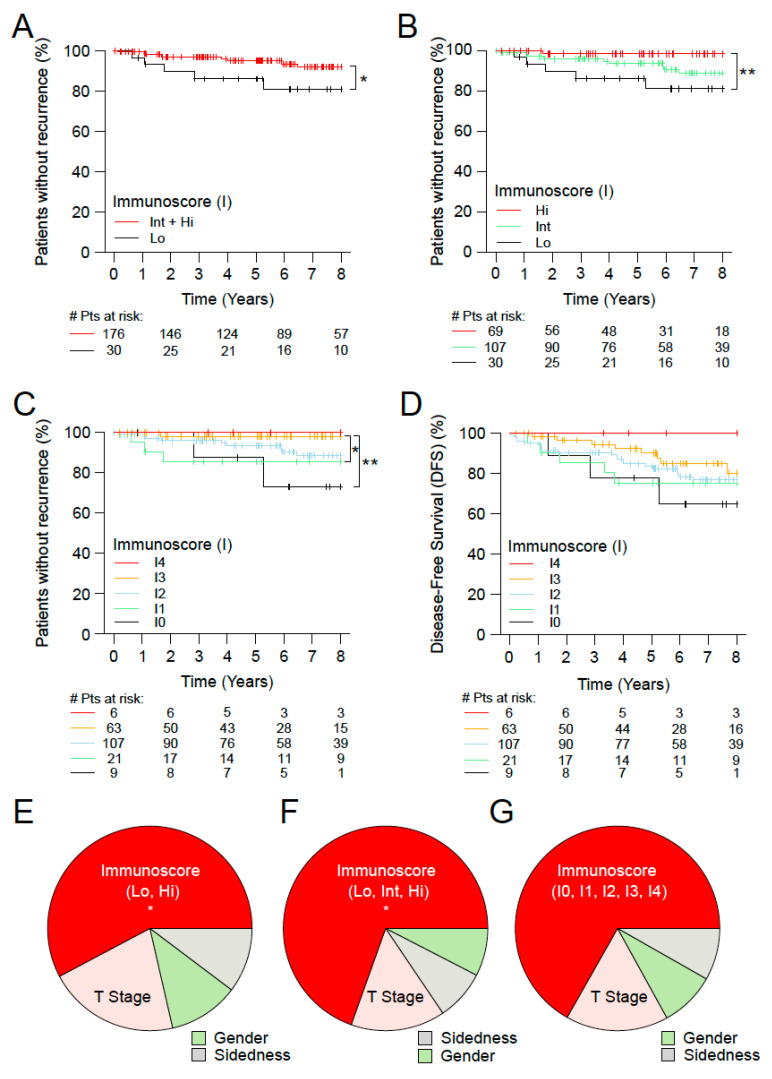
The impact of Immunoscore on MSS patients with Stage I colon cancer. Kaplan-Meier curves of Immunoscore (I) for TTR (**A**–**C**) and DFS (**D**) for Stage I MSS patients from cohorts 1 and 2. (**A**) Immunoscore two categories, Lo (0–25%, black) and Int + Hi (>25–100%, red), in Stage I MSS patients. (**B**) Immunoscore three categories, I Lo (0–25%, black), I Int (>25–70%, green) and I Hi (>70–100%, red), in Stage I MSS patients. (**C,D**) Immunoscore five categories, I0 (0–10%, black), I1 (>10–25%, green), I2 (>25–70%, azure), I3 (>70–95%, orange) and I4 (>95–100%, red), in Stage I MSS patients. Relative importance of each risk parameter to survival risk for TTR using the χ^2^ proportion test for clinical parameters and Immunoscore in two (**E**), three (**F**) and five categories (**G**). Significant logrank *p*-values are marked as, ** 0.001 < *p* ≤ 0.01, * 0.01 < *p* ≤ 0.05.

Additionally, the consensus Immunoscore was investigated in the subgroup of MSS Stage I CC patients. Furthermore, we aimed to evaluate in these patients (*n* = 206) the prognostic value of the Immunoscore in two categories, for TTR and DFS outcomes (Figure 4). The two category Immunoscore permitted to identify patients with significant differences in the clinical outcomes for TTR. Patients identified with Int + Hi Immunoscore were associated with a significant prolonged survival for TTR (5 years recurrence rate Int + Hi: 4.7%, Lo: 14.0%; unadjusted HR Int + Hi vs. Lo = 0.27 95% CI, 0.08–0.87; *p* = 0.0279, Figure 4A). In three categories, patients with high (I3-4) (33.5%), I2 (51.9%), and low (I0-1) (14.6%) The Immunoscore presented recurrence rates at 5 years of 1.7%, 6.5%, 14% and unadjusted HR Hi vs. Lo = l; 95% CI, 0.01–0.61 *p* = 0.0167; (Figure 4B and Table 1). In five categories, the Immunoscore discriminated further patients for TTR and DFS (Figure 4C,D). In multivariable analysis for TTR, the variable with the most important relative contribution to the risk (χ^2^) was the Immunoscore, (contribution to the risk of 57.8%, 69.5%, 66.8% for Immunoscore in 2, 3, 5 categories, respectively). T-Stage, gender and sidedness had modest contribution compared to the Immunoscore (Figure 4E–G and Table S4).

## 4. Discussion

The present study demonstrates the robustness of the consensus Immunoscore assay in stratifying, with high precision, Immunoscore-high and low risk patients, with significant differences in clinical outcomes. This work was complied within the Standards for Reporting of Diagnostic Accuracy (STARD) guidelines (Table S5). The prognostic impact of the tumor immune contexture [14,44], and the recent international implementation of the Immunoscore assay in Stage I/II/III CC [12], confirmed new capabilities and the reproducibility of image assessment software to quantify immune cells within tumors. Beyond the results obtained for all Stages I/II/III [12,15,21], for localized cancers [12,26], and metastatic diseases (Stage IV) [32,33,34,35,36,45], the relevance of the consensus Immunoscore in early-stage patients remained to be established.

The use of chemotherapy in Stage II CC patients is still controversial, and no biomarkers can robustly predict the likelihood of response to chemotherapy. Although no randomized trial has been conducted on high-risk Stage II to evaluate the usefulness of chemotherapy in that population, chemotherapy is commonly given [41,46]. Risk parameters are important criteria to decide whether or not to treat patients with chemotherapy. Here, we report the consensus Immunoscore as a powerful stratifier for Stage II patients, including Stage II, MSS Stage II, untreated Stage II, high-risk Stage II and T4 tumors. Importantly, within Stage II patients, the Immunoscore has the most important relative contribution to the risk of DFS (72%) compared to all other clinical parameters.

Our data show that despite the presence of high-risk features that usually trigger adjuvant treatment, when not treated with chemotherapy, a significant part of these patients (69.5%) with a high Immunoscore have a recurrence risk similar to the low-risk patients. Therefore, the Immunoscore test could be a good tool for adjuvant treatment decision in Stage II patients. This indicates that the major impact of the Immunoscore may be to classify patients into low- and high-risk groups, and identifies the need to perform randomized clinical trials, to evaluate treatment options for Stage II patients.

Stage I patients are typically considered as very low-risk patients. However, our data also support the usefulness of the Immunoscore to predict high-risk Stage I patients. The Immunoscore is a robust prognostic indicator of the risk of recurrence in Stage I CC. This risk assessment tool reliably identifies a subgroup of patients with an increased risk of relapse for whom a more intensive surveillance program after curative resection may be recommended. We previously reported an inverse correlation between pre-existing intratumoral adaptive immune cell densities, the Immunoscore, and tumor progression. In fact, the highest densities of adaptive immune cells were observed in the earliest tumors stages [21,47]. These indicated that adaptive immunity, including cytotoxic CD8 T-cells and helper CD4 T-cells with Th1 signature (IFNG, IL12, IRF1) [48], might arise before the carcinoma stage. We recently validated this hypothesis, showing adaptive immune infiltration, increased adaptive immunity, as well as immune escape mechanisms, including immune checkpoint, in pre-cancer lesions [49]. This opens the possibility to perform immunotherapy at the earliest stage of cancer, such as Stage I, and even during the carcinogenesis at a pre-cancer stage.

Several histologic factors have been proposed to evaluate the risk of lymph-node metastasis of early-stage colorectal cancer, including poorly differentiated tumors, positive lymphatic invasion (LI), positive venous emboli (VE), positive perineural invasion (PI), T4 tumors, and high-grade tumor budding [3,4,6]. Apart from its prognostic ability, the Immunoscore acts as a predictive factor of dissemination to metastasis [34]. We previously showed that the adaptive immunity and T-cells correlated with the absence of early-metastatic invasion (VELIPI) [25]. Recently, it was shown that the Immunoscore had a predictive value of response to chemotherapy for stage III patients in a randomized phase 3 clinical trial. High Immunoscore patients benefited from a longer duration of FOLFOX6 treatment. The Immunoscore also predicted patients who benefited from 6 months FOLFOX6 within low and high-risk pathological-stages [39]. Multiple therapies may rely on the pre-existing immune contexture [14,44,46,50,51,52,53,54,55,56,57]. Thus, Immunoscore evaluation may have important clinical consequences, for both early and late-stage colon cancer [30,31,58,59,60,61].

One constraint of this study might be the heterogeneity of the patient population coming from 13 different countries. However, such a non-randomized study also showed the robustness of the consensus Immunoscore assay across multiple patient care and ethnicities. We are looking now for a further validation of the Immunoscore assay in randomized clinical trials. This would be of high importance to evaluate the Immunoscore predictive potential for response to chemotherapy, as well as for other prognostic purposes.

## 5. Conclusions

The usefulness of the Immunoscore across all stages and within stage III CC has been recently reported [38,39,40,62]. The Immunoscore also has a broad applicability to other cancer types, since immune cells have a profound impact on survival for multiple cancers [13,44,63,64]. The latest edition of the WHO classification of CRC now recommends, for the first time, the inclusion of cytotoxic T-cells densities evaluated in the center and invasive margin of tumor, which is performed by the consensus Immunoscore. Furthermore, the Immunoscore was introduced into the 2020 European ESMO Clinical Practice Guidelines for CC and into the 2021 Pan-Asian adapted ESMO Clinical Practice Guidelines, to refine the prognosis and thus adjust the chemotherapy decision-making process [65,66]. The establishment of such an international consensus Immunoscore also argues for the revision of other cancer guidelines, such as NCCN, CAP, and AJCC/UICC-TNM, by introducing the consensus Immunoscore. In the present study, we demonstrated that the Immunoscore provides a powerful stratification method, based on immunity and not on tumor cell characteristics. This could help in classifying patients at different risks and help in directing the therapeutic strategy in early-stage colon cancers.

## 6. Patents

JG, FP, and BM have patents associated with the immune prognostic biomarkers. Immunoscore^®^ is a registered trademark owned by the National Institute of Health and Medical Research (INSERM) and licensed to Veracyte. Michael Roehrl is a member of the Scientific Advisory Boards of Azenta and Universal DX. All other authors declare no conflicts of interest.

## Figures and Tables

**Table 1:** Univariate Analysis (Cohort1+Cohort2).

STAGE I-II (Cohorts 1+2)									
Time to Recurrence (TTR)				Unadjusted Stratified by Center			Restricted Mean Survival Time (RMST)		
	No. of Patients (%)	Rate at 5 yr % (95% CI)	*p* Value *	Hazard Ratio (95% CI)	*p* Value **	C-Index (95% CI)	Months (95% CI)	Relative Months (95% CI)	*p* Value ***
**IS-2Level**						0.59 (0.52–0.66)			
0–25%	448 (23.8)	78.4 (74.4–82.6)		1.0 (reference)			208.1 (197.1–219)	0.0 (reference)	
25–100%	1437 (76.2)	90 (88.3–91.8)	<0.0001	0.43 (0.32–0.57)	<0.0001		240.9 (236.3–245.6)	32.9 (21–44.8)	<0.0001
**IS-3Level**						0.63 (0.53–0.72)			
0–25%	448 (23.8)	78.4 (74.4–82.6)		1.0 (reference)			208.1 (197.1–219)	0.0 (reference)	
25–70%	890 (47.2)	88.1 (85.7–90.4)	<0.0001	0.52 (0.39–0.7)	<0.0001		235.4 (229.1–241.7)	27.3 (14.7–40)	<0.0001
70–100%	547 (29)	93.4 (91.1–95.8)	<0.0001	0.27 (0.18–0.41)	<0.0001		250.5 (244.2–256.7)	42.4 (29.8–55)	<0.0001
**Disease free survival (DFS)**				**Unadjusted stratified by center**			**Restricted Mean Survival Time (RMST)**		
	**No. of patients (%)**	**Rate at 5 yr % (95% CI)**	*p* Value *	**Hazard ratio (95% CI)**	*p* Value **	**C-index (95% CI)**	**Months (95% CI)**	**Relative Months (95% CI)**	*p* Value ***
**IS-2Level**						0.54 (0.5–0.59)			
0–25%	448 (23.8)	67.3 (62.9–72)		1.0 (reference)			124.3 (111–137.6)	0.0 (reference)	
25–100%	1437 (76.2)	78 (75.7–80.3)	<0.0001	0.67 (0.56–0.79)	<0.0001		154.9 (146.6–163.1)	30.6 (14.9–46.3)	0.0001
**IS-3Level**						0.56 (0.5–0.61)			
0–25%	448 (23.8)	67.3 (62.9–72)		1.0 (reference)			124.3 (111–137.6)	0.0 (reference)	
25–70%	890 (47.2)	76.8 (73.9–79.8)	0.0004	0.72 (0.6–0.86)	0.0005		151.5 (141.3–161.7)	27.2 (10.4–44)	0.0015
70–100%	547 (29)	80 (76.4–83.7)	<0.0001	0.57 (0.45–0.71)	<0.0001		161.4 (147.2–175.6)	37.1 (17.7–56.6)	0.0002
**STAGE I, MSS (Cohorts 1+2)**									
**Time to recurrence (TTR)**				**Unadjusted stratified by center**			**Restricted Mean Survival Time (RMST)**		
	**No. of patients (%)**	**Rate at 5 yr % (95% CI)**	*p* Value *	**Hazard ratio (95% CI)**	*p* Value **	**C-index (95% CI)**	**Months (95% CI)**	**Relative Months (95% CI)**	*p* Value ***
**IS-2Level**						0.65 (0.48–0.82)			
0–25%	30 (14.6)	86 (74.2–99.7)		1.0 (reference)			156.4 (132.9–179.9)	0.0 (reference)	
25–100%	176 (85.4)	95.3 (92–98.8)	0.0427	0.27 (0.08–0.87)	0.0279		174.7 (168–181.4)	18.3 (−6.1–42.7)	0.1414
**IS-3Level**						0.72 (0.45–0.98)			
0–25%	30 (14.6)	86 (74.2–99.7)		1.0 (reference)			156.4 (132.9–179.9)	0.0 (reference)	
25–70%	107 (51.9)	93.5 (88.5–98.7)	0.2068	0.38 (0.12–1.22)	0.1047		170 (160.2–179.8)	13.6 (−11.8–39.1)	0.2939
70–100%	69 (33.5)	98.3 (95.1–100)	0.0056	0.07 (0.01–0.61)	0.0167		183.1 (177.7–188.5)	26.7 (2.6–50.8)	0.0296
**Disease free survival (DFS)**				**Unadjusted stratified by center**			**Restricted Mean Survival Time (RMST)**		
	**No. of patients (%)**	**Rate at 5 yr % (95% CI)**	*p* Value *	**Hazard ratio (95% CI)**	*p* Value **	**C-index (95% CI)**	**Months (95% CI)**	**Relative Months (95% CI)**	*p* Value ***
**IS-2Level**						0.55 (0.45–0.65)			
0–25%	30 (14.6)	76 (62–93.2)		1.0 (reference)			141.9 (115.9–168)	0.0 (reference)	
25–100%	176 (85.4)	86.5 (81.3–92.1)	0.2536	0.59 (0.27–1.3)	0.1876		148.9 (138–159.8)	7 (−21.2–35.2)	0.6280
**IS-3Level**						0.6 (0.45–0.75)			
0–25%	30 (14.6)	76 (62–93.2)		1.0 (reference)			141.9 (115.9–168)	0.0 (reference)	
25–70%	107 (51.9)	83.7 (76.6–91.4)	0.5450	0.69 (0.31–1.54)	0.3627		143.2 (128.9–157.5)	1.3 (−28.4–31)	0.9313
70–100%	69 (33.5)	91.1 (83.9–98.9)	0.0737	0.37 (0.13–1)	0.0511		158.8 (142.6–174.9)	16.8 (−13.8–47.5)	0.2816
**STAGE II, MSS (Cohorts 1+2)**									
**Time to recurrence (TTR)**				**Unadjusted stratified by center**			**Restricted Mean Survival Time (RMST)**		
	**No. of patients (%)**	**Rate at 5 yr % (95% CI)**	*p* Value *	**Hazard ratio (95% CI)**	*p* Value **	**C-index (95% CI)**	**Months (95% CI)**	**Relative Months (95% CI)**	*p* Value ***
**IS-2Level**						0.58 (0.5–0.66)			
0–25%	183 (28.1)	71.3 (64.7–78.6)		1.0 (reference)			159.9 (145.5–174.3)	0.0 (reference)	
25–100%	469 (71.9)	85.9 (82.6–89.3)	<0.0001	0.49 (0.36–0.66)	<0.0001		191.6 (184.5–198.7)	31.7 (15.6–47.8)	0.0001
**IS-3Level**						0.61 (0.51–0.7)			
0–25%	183 (28.1)	71.3 (64.7–78.6)		1.0 (reference)			159.9 (145.5–174.3)	0.0 (reference)	
25–70%	336 (51.5)	85.3 (81.4–89.4)	0.0001	0.56 (0.41–0.77)	0.0003		190 (181.5–198.5)	30.1 (13.4–46.8)	0.0004
70–100%	133 (20.4)	87.4 (81.3–93.9)	0.0007	0.33 (0.21–0.52)	<0.0001		195.8 (183.2–208.4)	35.9 (16.7–55.1)	0.0002
**Disease free survival (DFS)**				**Unadjusted stratified by center**			**Restricted Mean Survival Time (RMST)**		
	**No. of patients (%)**	**Rate at 5 yr % (95% CI)**	*p* Value *	**Hazard ratio (95% CI)**	*p* Value **	**C-index (95% CI)**	**Months (95% CI)**	**Relative Months (95% CI)**	*p* Value ***
**IS-2Level**						0.54 (0.49–0.59)			
0–25%	183 (28.1)	60.6 (53.8–68.3)		1.0 (reference)			113.2 (96.5–129.9)	0.0 (reference)	
25–100%	469 (71.9)	75.8 (71.9–79.9)	<0.0001	0.71 (0.58–0.85)	0.0003		148 (137.8–158.3)	34.8 (15.2–54.4)	0.0005
**IS-3Level**						0.55 (0.49–0.61)			
0–25%	183 (28.1)	60.6 (53.8–68.3)		1.0 (reference)			113.2 (96.5–129.9)	0.0 (reference)	
25–70%	336 (51.5)	75.5 (70.9–80.4)	0.0001	0.75 (0.61–0.91)	0.0044		149.2 (137.2–161.3)	36 (15.5–56.6)	0.0006
70–100%	133 (20.4)	76.5 (69.1–84.6)	0.0022	0.63 (0.49–0.8)	0.0002		147 (127.9–166.1)	33.8 (8.4–59.1)	0.0090

* Logrank *P* Value. ** Wald *p* Value stratified by participating center. *** Restricted Mean Survival Time (RMST) *p* value. MSS: proficient Mismatch repair (pMMR).

## Data Availability

Data are available upon request to the corresponding author.

## Supplementary Materials

The following supporting information can be downloaded at: https://www.mdpi.com/article/10.3390/cancers15020418/s1, Figure S1: The impact of Immunoscore on the survival of patients from cohorts 1+2; Figure S2: The impact of Immunoscore on patients with Stage II colon cancer; Figure S3: The impact of Immunoscore on patients with high-risk Stage II colon cancer; Figure S4: The impact of Immunoscore on patients with Stage I colon cancer; Table S1: Demographic distribution; Table S2: Univariate analysis STAGE I-II; Table S3: Univariate analysis STAGE II; Table S4: Multivariable analysis (Cohorts 1+2); Table S5: STARD checklist.
