# Supplementary material for: Multicenter International Study of the Consensus Immunoscore for the Prediction of Relapse and Survival in Early-Stage Colon Cancer
Source: Cancers (Basel). 2023 Jan 8;15(2):418. doi: 10.3390/cancers15020418 (PMC9856473; doi:10.3390/cancers15020418)
Supplement: Supplementary file 1 [file cancers-15-00418-s001.zip › cancers-1964145-supplementary.pdf]

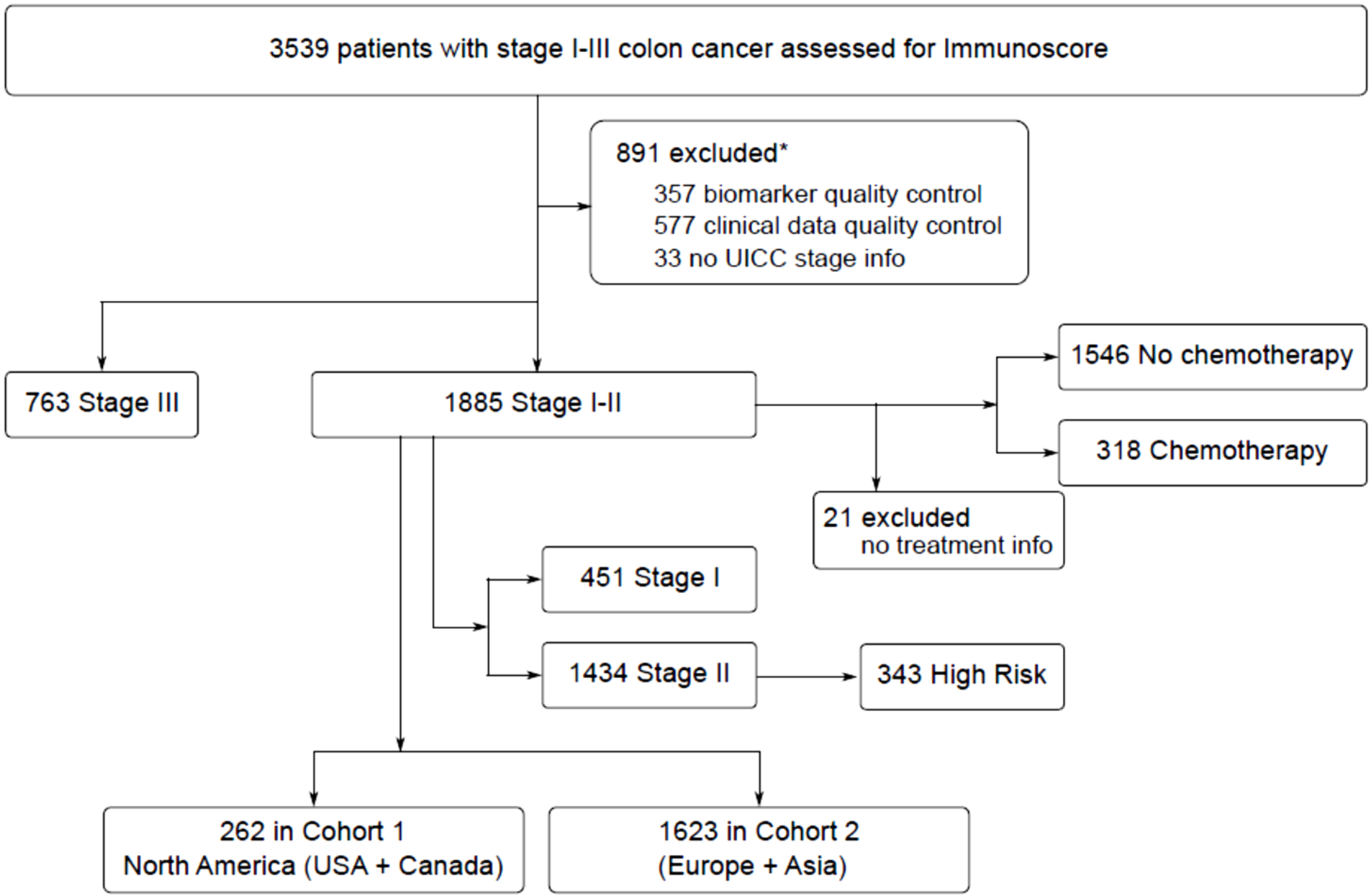
3539 patients with stage I-III colon cancer assessed for Immunoscore
891 excluded*
357 biomarker quality control
577 clinical data quality control
33 no UICC stage info
763 Stage III
1885 Stage I-II
1546 No chemotherapy
318 Chemotherapy
21 excluded
no treatment info
451 Stage I
1434 Stage II
343 High Risk
262 in Cohort 1
North America (USA + Canada)
1623 in Cohort 2
(Europe + Asia)

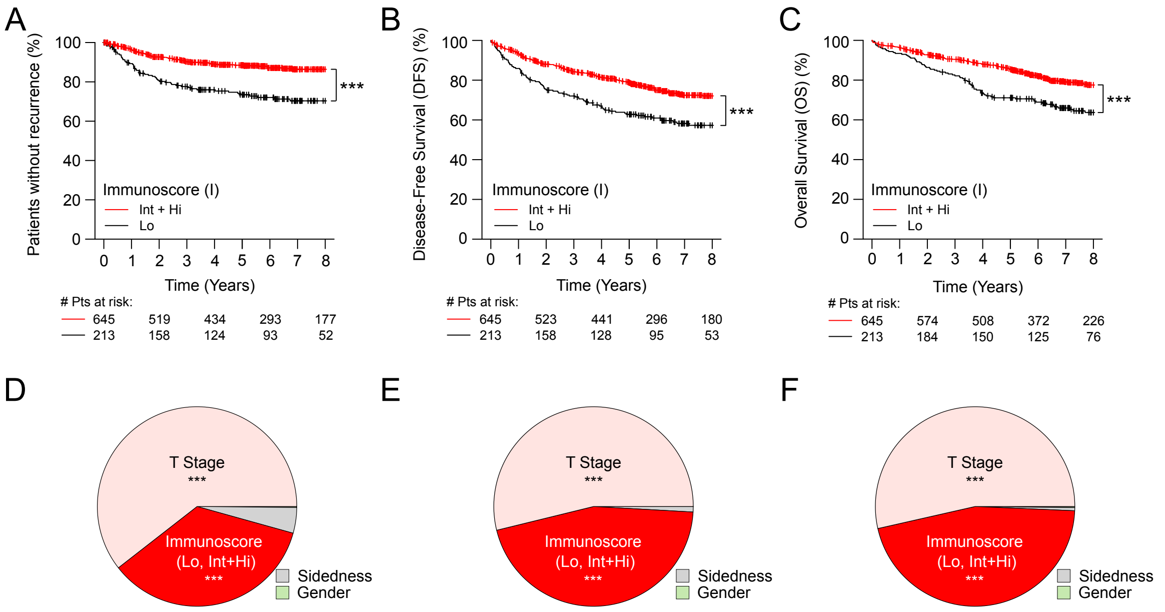

Figure S1. The impact of Immunoscore on the survival of patients from cohorts 1+2 Kaplan-Meier curves of Immunoscore (I) are shown for TTR **(A)**, DFS **(B)** and OS **(C)** for MSS Stage I/II patients from cohort 1+2 **(A-C)**. Immunoscore two categories, I Lo (0 -25%, black), I Int+Hi (>25 -100%, red), is illustrated. Relative importance of each risk parameter to survival risk for TTR **(D)**, DFS **(E)** and OS **(F)** using the χ^2 proportion test for clinical parameters and Immunoscore corresponding to panel A-C. Significant logrank *P*-values are marked as *** p< 0.001.

Figure S1

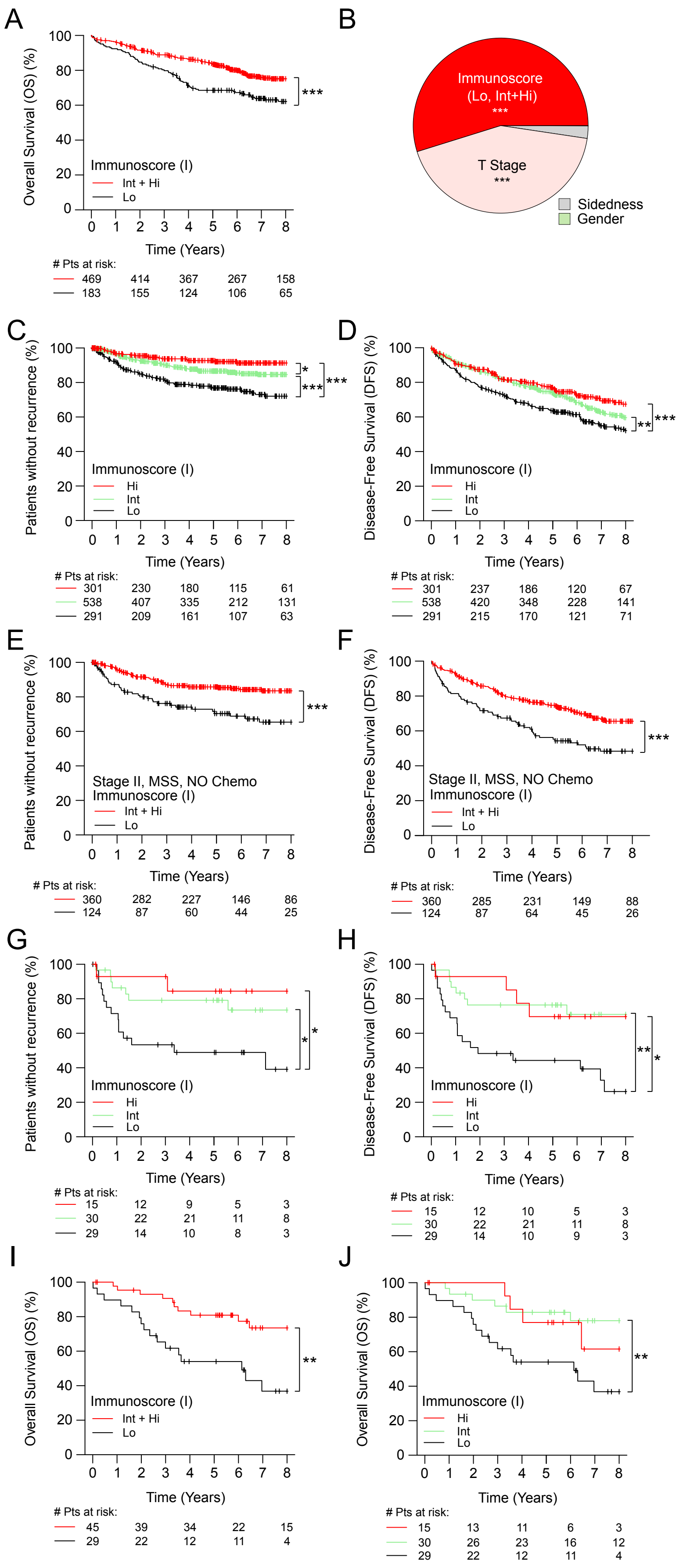
A
Overall Survival (OS) (%)
Immunoscore (I)
Int + Hi
Lo
Time (Years)
Pts at risk:
469 414 367 267 158
183 155 124 106 65
B
Immunoscore
(Lo, Int+Hi)
T Stage
Sidedness
Gender
C
Patients without recurrence (%)
Immunoscore (I)
Hi
Int
Lo
Time (Years)
Pts at risk:
301 230 180 115 61
538 407 335 212 131
291 209 161 107 63
D
Disease-Free Survival (DFS) (%)
Immunoscore (I)
Hi
Int
Lo
Time (Years)
Pts at risk:
301 237 186 120 67
538 420 348 228 141
291 215 170 121 71
E
Patients without recurrence (%)
Stage II, MSS, NO Chemo
Immunoscore (I)
Int + Hi
Lo
Time (Years)
Pts at risk:
360 282 227 146 86
124 87 60 44 25
F
Disease-Free Survival (DFS) (%)
Stage II, MSS, NO Chemo
Immunoscore (I)
Int + Hi
Lo
Time (Years)
Pts at risk:
360 285 231 149 88
124 87 64 45 26
G
Patients without recurrence (%)
Immunoscore (I)
Hi
Int
Lo
Time (Years)
Pts at risk:
15 12 9 5 3
30 22 21 11 8
29 14 10 8 3
H
Disease-Free Survival (DFS) (%)
Immunoscore (I)
Hi
Int
Lo
Time (Years)
Pts at risk:
15 12 10 5 3
30 22 21 11 8
29 14 10 9 3
I
Overall Survival (OS) (%)
Immunoscore (I)
Int + Hi
Lo
Time (Years)
Pts at risk:
45 39 34 22 15
29 22 12 11 4
J
Overall Survival (OS) (%)
Immunoscore (I)
Hi
Int
Lo
Time (Years)
Pts at risk:
15 13 11 6 3
30 26 23 16 12
29 22 12 11 4

Figure S2

Figure S2. The impact of Immunoscore on patients with Stage II colon cancer.

Kaplan-Meier curves of Immunoscore (I) are shown for OS **(A, I, J)**, TTR **(C, E, G)** and DFS **(D, F, H)** for subgroups of Stage II patients from cohorts 1 and 2. **(A)** Kaplan-Meier curves for OS are shown for Immunoscore two categories: Lo (0-25%, black) and Int+Hi (>25-100%, red) in MSS Stage II patients. **(B)** Relative importance of each risk parameter to survival risk for OS using the χ^2 proportion test for clinical parameters and Immunoscore corresponding to panel A. **(C, D)** Immunoscore three categories, I Lo (0-25%, black), I Int (>25-70%, green) and I Hi (>70-100%, red), in Stage II patients who did not receive adjuvant chemotherapy for TTR **(C)** and DFS **(D)**. **(E, F)** Immunoscore two categories: Lo (0-25%, black) and Int+Hi (>25-100%, red) in MSS Stage II patients without chemotherapy treatment. **(G, H)** Immunoscore three categories in high-risk Stage II patients for TTR **(G)** and DFS **(H)**. **(I, J)** Kaplan-Meier curves for OS are shown for Immunoscore two categories **(I)** and three categories **(J)** in high-risk Stage II patients. Significant logrank *P*-values are marked as *** $p < 0.001$, ** $0.001 < p \leq 0.01$, * $0.01 < p \leq 0.05$.

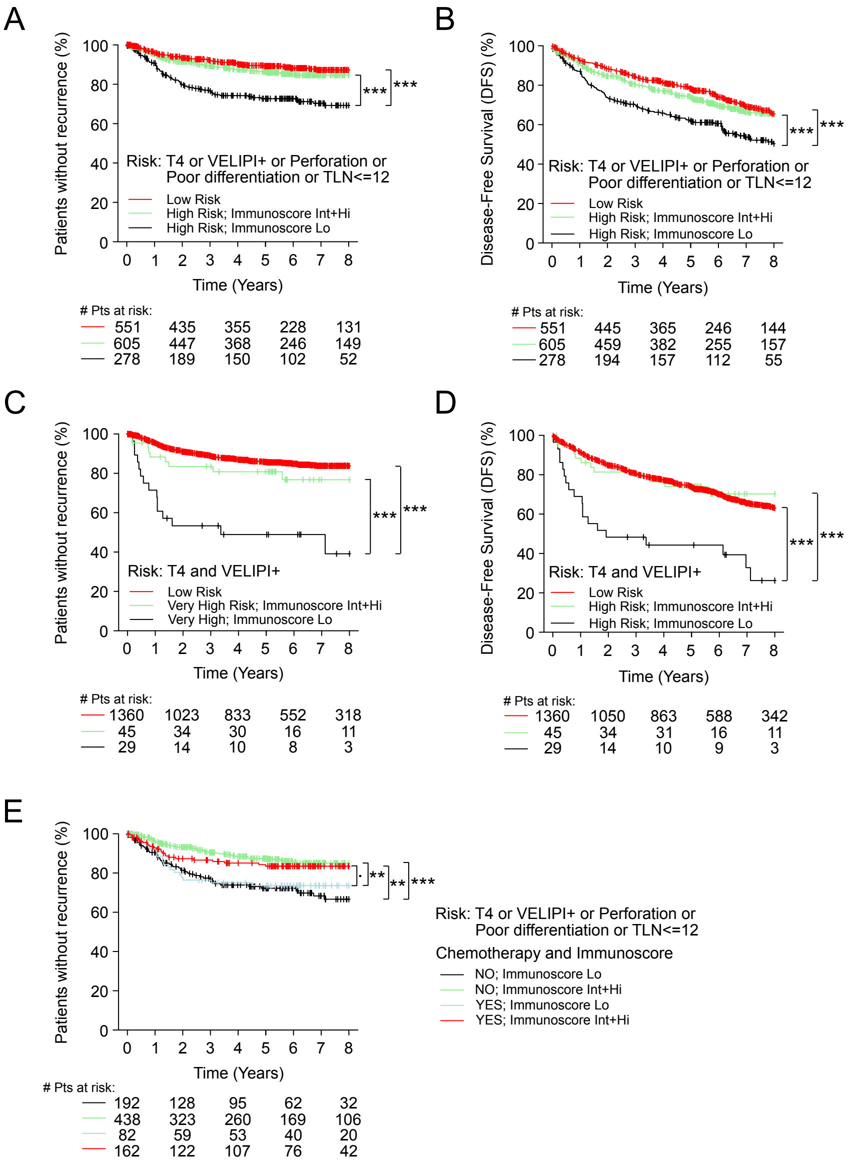

Figure S3. The impact of Immunoscore on patients with high -risk Stage II colon cancer. Kaplan-Meier curves of Immunoscore (I) three categories, I Lo (0-25%, black), I Int (>25-70%, green) and I Hi (>70-100%, red) are shown for TTR **(A, C)** and DFS **(B, D)** for subgroups of Stage II patients from cohorts 1 and 2. **(A, B)** high-risk Stage II patients: T4 or VELIPI+ or perforation or poor differentiation or TLN≤12 . **(C, D)** very high-risk Stage II patients: T4 and VELIPI+. **(E)** High risk Stage II patients, Immunoscore two categories Lo (0 -25%), I Int+Hi (>25 -100%) and chemotherapy (YES/NO) are shown for TTR. Significant logrank *P*-values are marked as *** $p < 0.001$, ** $0.001 < p \leq 0.01$.

Figure S3

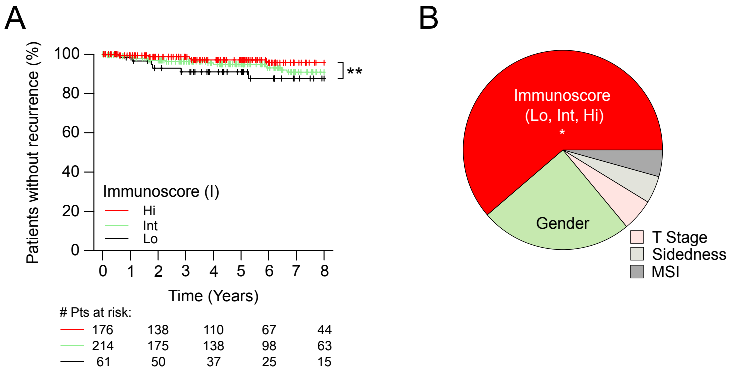

Figure S4. The impact of Immunoscore on patients with Stage I colon cancer. **(A)** Kaplan-Meier curves for TTR of Immunoscore (I) three categories, I Lo (0 -25%, black), I Int (>25 -70%, green) and I Hi (>70 -100%, red), in Stage I patients from cohorts 1 and 2. **(B)** Relative importance of each risk parameter to survival risk for TTR using the χ^2 proportion test for clinical parameters and Immunoscore in three categories corresponding to panel A . Significant logrank *P*-values are marked as ** $0.001 < p \leq 0.01$, * $0.01 < p \leq 0.05$.

Figure S4

Table S1. Demographic distribution.	North-America Cohort 1 (N=262)	Europe & Asia Cohort 2 (N=1623)	Total Cohorts 1+2 (N=1885)	Fisher's exact test P-value Cohorts 1 vs. 2	STAGE I Cohorts 1+2 (N=451)	STAGE II Cohorts 1+2 (N=1434)	STAGE II very high risk Cohorts 1+2 (N=74)
**Age**				0.1517 *			
**N**	262	1623	1885		451	1434	74
**Mean (SD)**	66.9 (12.5)	68.1 (12.2)	67.9 (12.2)		67.3 (12.5)	68.2 (12.1)	66.4 (11.4)
**Range**	(23-92)	(21-101)	(21-101)		(22-95)	(21-101)	(40-92)
**Gender**				0.7389			
**Male**	135 (51.5%)	857 (52.8%)	992 (52.6%)		238 (52.8%)	754 (52.6%)	34 (45.9%)
**Female**	127 (48.5%)	766 (47.2%)	893 (47.4%)		213 (47.2%)	680 (47.4%)	40 (55.4%)
**T-stage**				<0.0001			
**T1**	36 (13.7%)	80 (4.9%)	116 (6.2%)		116 (25.7%)	0 (0.0%)	0 (0.0%)
**T2**	66 (25.2%)	269 (16.6%)	335 (17.8%)		335 (74.3%)	0 (0.0%)	0 (0.0%)
**T3**	143 (54.6%)	1083 (66.7%)	1226 (65%)		0 (0.0%)	1226 (85.5%)	0 (0.0%)
**T4**	17 (6.5%)	191 (11.8%)	208 (11%)		0 (0.0%)	208 (14.5%)	74 (100%)
**N-stage**							
**N0**	262 (100.0%)	1623 (100.0%)	1885 (100.0%)		451 (100%)	1434 (100.0%)	74 (100%)
**N+**	0 (0.0%)	0 (0.0%)	0 (0.0%)		0 (0.0%)	0 (0.0%)	0 (0.0%)
**Total lymnodes examined**				0.8710 *			
**N**	262	1623	1885		451	1434	74
**Mean (SD)**	19.1 (12.9)	18.9 (13.9)	19.0 (13.8)		15.2 (10.4)	20.1 (14.5)	21.3 (14.7)
**Range**	(1-108)	(1-145)	(1-145)		(1-62)	(1-145)	(1-62)
**Not Available**	8 (3.1%)	0 (0.0%)	0 (0.04%)		4 (0.9%)	4 (0.3%)	0 (0.0%)
**M-stage**							
**M0**	262 (100.0%)	1623 (100.0%)	1885 (100.0%)		451 (100%)	1434 (100.0%)	74 (100%)
**AJCC/UICC-TNM Composite Stage**				<0.0001			
**I**	102 (38.9%)	349 (21.5%)	451 (23.9%)		451 (100%)	0 (0.0%)	0 (0.0%)
**II**	160 (61.1%)	1274 (78.5%)	1434 (76.1%)		0 (0.0%)	1434 (100.0%)	74 (100%)
**Differentiation Grade**				<0.0001			
**Well**	32 (12.2%)	434 (26.7%)	466 (24.7%)		143 (31.7%)	323 (22.5%)	14 (18.9%)
**Moderate**	202 (77.1%)	700 (43.1%)	902 (47.9%)		222 (49.2%)	680 (47.4%)	42 (56.8%)
**Poor**	25 (9.5%)	141 (8.7%)	166 (8.8%)		19 (4.2%)	147 (10.3%)	8 (10.8%)
**Not Available**	3 (1.1%)	348 (21.4%)	351 (18.6%)		67 (14.9%)	284 (19.8%)	10 (13.5%)
**Post-Operative Chemotherapy (Yes/No)**				0.07509			
**No**	226 (86.3%)	1320 (81.3%)	1546 (82%)		416 (92.2%)	1130 (78.8%)	36 (48.6%)
**Yes**	34 (13%)	284 (17.5%)	318 (16.9%)		27 (6%)	291 (20.3%)	38 (51.4%)
**Not Available**	2 (0.8%)	19 (1.2%)	21 (1.1%)		8 (1.8%)	13 (0.9%)	0 (0.0%)
**Proximal vs. Distal Primary (Tumor)**				0.0618			
**Proximal**	147 (56.1%)	798 (49.2%)	945 (50.1%)		208 (46.1%)	737 (51.4%)	37 (50%)
**Distal**	115 (43.9%)	810 (49.9%)	925 (49.1%)		240 (53.2%)	685 (47.8%)	36 (48.6%)
**Not Available**	0 (0%)	15 (0.9%)	15 (0.8%)		3 (0.7%)	12 (0.8%)	1 (1.4%)
**MSI Status (Derived)**				0.5934			
**pMMR**	119 (45.4%)	739 (45.5%)	858 (45.5%)		206 (45.7%)	652 (45.5%)	33 (44.6%)
**dMMR**	35 (13.4%)	193 (11.9%)	228 (12.1%)		39 (8.6%)	189 (13.2%)	12 (16.2%)
**Not Available**	108 (41.2%)	691 (42.6%)	799 (42.4%)		206 (0.7%)	593 (41.4%)	29 (39.2%)

* T test P-value. MSI: deficient Mismatch repair (dMMR), MSS: proficient Mismatch repair (pMMR).

Table S2. Univariate analysis STAGE I-II

STAGE I-II, North-America (Cohort 1)

**Time to recurrence (TTR)**				**Unadjusted stratified by center**			**Restricted Mean Survival Time (RMST)**		
	**No. of patients (%)**	**Rate at 5 yr % (95% CI)**	**P value***	**Hazard ratio (95% CI)**	**P value****	**C-index (95% CI)**	**Months (95% CI)**	**Relative Months (95% CI)**	**P value*****
**IS-2Level**						0.62 (0.55-0.68)			
**0-25%**	21 (8)	60 (41.6-86.5)		1.0 (reference)			101.6 (72.3-130.9)	0.0 (reference)	
**25-100%**	241 (92)	91.4 (87.5-95.4)	<.0001	0.19 (0.08-0.43)	<.0001		142.6 (137.2-148.1)	41.1 (11.2-70.9)	0.0070
**IS-3Level**						0.69 (0.56-0.82)			
**0-25%**	21 (8)	60 (41.6-86.5)		1.0 (reference)			101.6 (72.3-130.9)	0.0 (reference)	
**25-70%**	143 (54.6)	88.3 (82.7-94.3)	0.0014	0.27 (0.11-0.62)	0.0023		138 (130.1-146)	36.4 (6-66.8)	0.0189
**70-100%**	98 (37.4)	96.3 (92.3-100)	<.0001	0.07 (0.02-0.27)	0.0001		144.8 (139.4-150.2)	46.6 (18-75.3)	0.0014

**Disease free survival (DFS)**				**Unadjusted stratified by center**			**Restricted Mean Survival Time (RMST)**		
	**No. of patients (%)**	**Rate at 5 yr % (95% CI)**	**P value***	**Hazard ratio (95% CI)**	**P value****	**C-index (95% CI)**	**Months (95% CI)**	**Relative Months (95% CI)**	**P value*****
**IS-2Level**						0.54 (0.5-0.58)			
**0-25%**	21 (8)	60.6 (42.5-86.5)		1.0 (reference)			96.5 (67.4-125.6)	0.0 (reference)	
**25-100%**	241 (92)	83.1 (78.2-88.2)	0.0646	0.48 (0.24-0.97)	0.0399		117.9 (110.2-125.6)	21.4 (-8.7-51.5)	0.1636
**IS-3Level**						0.54 (0.45-0.62)			
**0-25%**	21 (8)	60.6 (42.5-86.5)		1.0 (reference)			96.5 (67.4-125.6)	0.0 (reference)	
**25-70%**	143 (54.6)	83.2 (77-89.9)	0.0425	0.45 (0.22-0.95)	0.0359		121.5 (111.7-131.2)	25 (-5.7-55.7)	0.1102
**70-100%**	98 (37.4)	82.9 (75.3-91.2)	0.1735	0.51 (0.24-1.09)	0.0835		110.8 (99.1-122.5)	17.4 (-12.8-47.7)	0.2580

STAGE I-II, Europe & Asia (Cohort 2)

**Time to recurrence (TTR)**				**Unadjusted stratified by center**			**Restricted Mean Survival Time (RMST)**		
	**No. of patients (%)**	**Rate at 5 yr % (95% CI)**	**P value***	**Hazard ratio (95% CI)**	**P value****	**C-index (95% CI)**	**Months (95% CI)**	**Relative Months (95% CI)**	**P value*****
**IS-2Level**						0.59 (0.52-0.66)			
**0-25%**	427 (26.3)	79.4 (75.4-83.7)		1.0 (reference)			210.1 (198.9-221.2)	0.0 (reference)	
**25-100%**	1196 (73.7)	89.8 (87.9-91.7)	<.0001	0.47 (0.35-0.63)	<.0001		240.2 (235.1-245.3)	30.2 (17.9-42.4)	<.0001
**IS-3Level**						0.62 (0.53-0.71)			
**0-25%**	427 (26.3)	79.4 (75.4-83.7)		1.0 (reference)			210.1 (198.9-221.2)	0.0 (reference)	
**25-70%**	747 (46)	88 (85.5-90.6)	<.0001	0.55 (0.41-0.76)	0.0002		235.4 (228.5-242.3)	25.3 (12.2-38.4)	0.0001
**70-100%**	449 (27.7)	92.8 (90.1-95.5)	<.0001	0.31 (0.2-0.49)	<.0001		248.6 (241.3-255.8)	38.5 (25.2-51.8)	<.0001

**Disease free survival (DFS)**				**Unadjusted stratified by center**			**Restricted Mean Survival Time (RMST)**		
	**No. of patients (%)**	**Rate at 5 yr % (95% CI)**	**P value***	**Hazard ratio (95% CI)**	**P value****	**C-index (95% CI)**	**Months (95% CI)**	**Relative Months (95% CI)**	**P value*****
**IS-2Level**						0.54 (0.5-0.59)			
**0-25%**	427 (26.3)	67.6 (63.1-72.4)		1.0 (reference)			124.2 (110.8-137.7)	0.0 (reference)	
**25-100%**	1196 (73.7)	77 (74.5-79.6)	<.0001	0.68 (0.57-0.81)	<.0001		153.4 (144.8-162.1)	29.2 (13.2-45.2)	0.0003
**IS-3Level**						0.56 (0.5-0.61)			
**0-25%**	427 (26.3)	67.6 (63.1-72.4)		1.0 (reference)			124.2 (110.8-137.7)	0.0 (reference)	
**25-70%**	747 (46)	75.6 (72.4-78.9)	0.0044	0.75 (0.62-0.9)	0.0026		147.9 (137.4-158.4)	23.7 (6.6-40.7)	0.0066
**70-100%**	449 (27.7)	79.3 (75.3-83.5)	<.0001	0.55 (0.44-0.7)	<.0001		164.5 (149.3-179.8)	40.3 (20-60.6)	0.0001

STAGE I-II (Cohorts 1+2)

**Time to recurrence (TTR)**				**Unadjusted stratified by center**			**Restricted Mean Survival Time (RMST)**		
	**No. of patients (%)**	**Rate at 5 yr % (95% CI)**	**P value***	**Hazard ratio (95% CI)**	**P value****	**C-index (95% CI)**	**Months (95% CI)**	**Relative Months (95% CI)**	**P value*****
**IS-2Level**						0.59 (0.52-0.66)			
**0-25%**	448 (23.8)	78.4 (74.4-82.6)		1.0 (reference)			208.1 (197.1-219)	0.0 (reference)	
**25-100%**	1437 (76.2)	90 (88.3-91.8)	<.0001	0.43 (0.32-0.57)	<.0001		240.9 (236.3-245.6)	32.9 (21-44.8)	<.0001
**IS-3Level**						0.63 (0.53-0.72)			
**0-25%**	448 (23.8)	78.4 (74.4-82.6)		1.0 (reference)			208.1 (197.1-219)	0.0 (reference)	
**25-70%**	890 (47.2)	88.1 (85.7-90.4)	<.0001	0.52 (0.39-0.7)	<.0001		235.4 (229.1-241.7)	27.3 (14.7-40)	<.0001
**70-100%**	547 (29)	93.4 (91.1-95.8)	<.0001	0.27 (0.18-0.41)	<.0001		250.5 (244.2-256.7)	42.4 (29.8-55)	<.0001

**Disease free survival (DFS)**				**Unadjusted stratified by center**			**Restricted Mean Survival Time (RMST)**		
	**No. of patients (%)**	**Rate at 5 yr % (95% CI)**	**P value***	**Hazard ratio (95% CI)**	**P value****	**C-index (95% CI)**	**Months (95% CI)**	**Relative Months (95% CI)**	**P value*****
**IS-2Level**						0.54 (0.5-0.59)			
**0-25%**	448 (23.8)	67.3 (62.9-72)		1.0 (reference)			124.3 (111-137.6)	0.0 (reference)	
**25-100%**	1437 (76.2)	78 (75.7-80.3)	<.0001	0.67 (0.56-0.79)	<.0001		154.9 (146.6-163.1)	30.6 (14.9-46.3)	0.0001
**IS-3Level**						0.56 (0.5-0.61)			
**0-25%**	448 (23.8)	67.3 (62.9-72)		1.0 (reference)			124.3 (111-137.6)	0.0 (reference)	
**25-70%**	890 (47.2)	76.8 (73.9-79.8)	0.0004	0.72 (0.6-0.86)	0.0005		151.5 (141.3-161.7)	27.2 (10.4-44)	0.0015
**70-100%**	547 (29)	80 (76.4-83.7)	<.0001	0.57 (0.45-0.71)	<.0001		161.4 (147.2-175.6)	37.1 (17.7-56.6)	0.0002

STAGE I-II, MSS (Cohorts 1+2)

**Time to recurrence (TTR)**				**Unadjusted stratified by center**			**Restricted Mean Survival Time (RMST)**		
	**No. of patients (%)**	**Rate at 5 yr % (95% CI)**	**P value***	**Hazard ratio (95% CI)**	**P value****	**C-index (95% CI)**	**Months (95% CI)**	**Relative Months (95% CI)**	**P value*****
**IS-2Level**						0.59 (0.52-0.66)			
**0-25%**	213 (24.8)	73.5 (67.5-80)		1.0 (reference)			163.8 (150.7-176.8)	0.0 (reference)	
**25-100%**	645 (75.2)	88.5 (85.9-91.1)	<.0001	0.43 (0.32-0.57)	<.0001		196.2 (190.5-201.8)	32.4 (18.1-46.7)	<.0001
**IS-3Level**						0.63 (0.53-0.72)			
**0-25%**	213 (24.8)	73.5 (67.5-80)		1.0 (reference)			163.8 (150.7-176.8)	0.0 (reference)	
**25-70%**	443 (51.6)	87.3 (84-90.6)	<.0001	0.52 (0.39-0.7)	<.0001		192.9 (185.8-200.1)	29.2 (14.2-44.1)	0.0001
**70-100%**	202 (23.5)	91.1 (86.9-95.5)	<.0001	0.27 (0.18-0.41)	<.0001		203.8 (195-212.5)	40 (24.3-55.8)	<.0001

**Disease free survival (DFS)**				**Unadjusted stratified by center**			**Restricted Mean Survival Time (RMST)**		
	**No. of patients (%)**	**Rate at 5 yr % (95% CI)**	**P value***	**Hazard ratio (95% CI)**	**P value****	**C-index (95% CI)**	**Months (95% CI)**	**Relative Months (95% CI)**	**P value*****
**IS-2Level**						0.54 (0.5-0.59)			
**0-25%**	213 (24.8)	62.8 (56.5-69.8)		1.0 (reference)			116.3 (100.4-132.3)	0.0 (reference)	
**25-100%**	645 (75.2)	78.7 (75.4-82)	<.0001	0.67 (0.56-0.79)	<.0001		155.1 (146.5-163.6)	38.7 (20.6-56.8)	<.0001
**IS-3Level**						0.56 (0.5-0.61)			
**0-25%**	213 (24.8)	62.8 (56.5-69.8)		1.0 (reference)			116.3 (100.4-132.3)	0.0 (reference)	
**25-70%**	443 (51.6)	77.5 (73.5-81.6)	<.0001	0.72 (0.6-0.86)	0.0005		153 (142.6-163.4)	36.7 (17.6-55.7)	0.0002
**70-100%**	202 (23.5)	81.3 (75.7-87.4)	<.0001	0.57 (0.45-0.71)	<.0001		160.1 (145-175.2)	43.7 (21.8-65.7)	<.0001

**Overall survival (OS)**				**Unadjusted stratified by center**			**Restricted Mean Survival Time (RMST)**		
	**No. of patients (%)**	**Rate at 5 yr % (95% CI)**	**P value***	**Hazard ratio (95% CI)**	**P value****	**C-index (95% CI)**	**Months (95% CI)**	**Relative Months (95% CI)**	**P value*****
**IS-2Level**									
**0-25%**	213 (24.8)	71.1 (65.2-77.5)		1.0 (reference)			171.9 (151.3-192.5)	0.0 (reference)	
**25-100%**	645 (75.2)	85.4 (82.6-88.2)	<.0001				206 (193-219)	34.1 (9.7-58.5)	0.0061
**IS-3Level**									
**0-25%**	213 (24.8)	71.1 (65.2-77.5)		1.0 (reference)			171.9 (151.3-192.5)	0.0 (reference)	
**25-70%**	443 (51.6)	84.5 (81.1-88)	0.0003				206.3 (190.2-222.5)	34.4 (8.2-60.6)	0.0101
**70-100%**	202 (23.5)	87.4 (82.7-92.4)	0.0010				206.7 (184.4-229)	34.8 (4.4-65.2)	0.0247

* Logrank P Value. ** Wald P Value stratified by participating center. *** Restricted Mean Survival Time (RMST) P value

Table S3. Univariate analysis STAGE II

STAGE II, Without Chemotherapy (Cohorts 1+2)

Time to recurrence (TTR)	No. of patients (%)	Rate at 5 yr % (95% CI)	P value*	Unadjusted stratified by center<br>Hazard ratio (95% CI)	P value**	C-index (95% CI)	Restricted Mean Survival Time (RMST)<br>Months (95% CI)	Relative Months (95% CI)	P value***
**IS-2Level**						0.59 (0.51-0.68)			
**0-25%**	291 (25.8)	76.8 (71.7-82.3)		1.0 (reference)			202.3 (187.9-216.6)	0.0 (reference)	
**25-100%**	839 (74.2)	88.7 (86.4-91.1)	<.0001	0.44 (0.32-0.62)	<.0001		237.7 (231.4-244.1)	35.5 (19.8-51.2)	<.0001
**IS-3Level**						0.62 (0.52-0.73)			
**0-25%**	291 (25.8)	76.8 (71.7-82.3)		1.0 (reference)			202.3 (187.9-216.6)	0.0 (reference)	
**25-70%**	538 (47.6)	86.6 (83.4-89.9)	0.0001	0.52 (0.36-0.74)	0.0003		232.3 (223.9-240.7)	30 (13.4-46.7)	0.0004
**70-100%**	301 (26.6)	92.7 (89.4-96)	<.0001	0.3 (0.18-0.5)	<.0001		248 (239.1-256.9)	45.8 (28.9-62.7)	<.0001

Disease free survival (DFS)	No. of patients (%)	Rate at 5 yr % (95% CI)	P value*	Unadjusted stratified by center<br>Hazard ratio (95% CI)	P value**	C-index (95% CI)	Restricted Mean Survival Time (RMST)<br>Months (95% CI)	Relative Months (95% CI)	P value***
**IS-2Level**						0.54 (0.49-0.59)			
**0-25%**	291 (25.8)	63.6 (58.2-69.6)		1.0 (reference)			112.9 (98.4-127.4)	0.0 (reference)	
**25-100%**	839 (74.2)	74.8 (71.8-78)	0.0004	0.7 (0.57-0.86)	0.0007		141.2 (131.2-151.3)	28.3 (10.7-46)	0.0016
**IS-3Level**						0.55 (0.48-0.61)			
**0-25%**	291 (25.8)	63.6 (58.2-69.6)		1.0 (reference)			112.9 (98.4-127.4)	0.0 (reference)	
**25-70%**	538 (47.6)	74 (70.1-78)	0.0031	0.74 (0.59-0.92)	0.0060		139 (127-151)	26.1 (7.3-44.9)	0.0064
**70-100%**	301 (26.6)	76.5 (71.5-81.8)	0.0008	0.64 (0.49-0.83)	0.0008		145.8 (127.5-164.2)	33 (9.6-56.3)	0.0057

STAGE II, MSS, Without Chemotherapy (Cohorts 1+2)

Time to recurrence (TTR)	No. of patients (%)	Rate at 5 yr % (95% CI)	P value*	Unadjusted stratified by center<br>Hazard ratio (95% CI)	P value**	C-index (95% CI)	Restricted Mean Survival Time (RMST)<br>Months (95% CI)	Relative Months (95% CI)	P value***
**IS-2Level**						0.57 (0.51-0.63)			
**0-25%**	124 (25.6)	70.3 (62-79.7)		1.0 (reference)			155 (136.3-173.6)	0.0 (reference)	
**25-100%**	360 (74.4)	85.7 (82-89.7)	0.0001	0.43 (0.27-0.67)	0.0002		190.4 (182-198.7)	35.4 (15-55.8)	0.0007
**IS-3Level**						0.59 (0.53-0.65)			
**0-25%**	124 (25.6)	70.3 (62-79.7)		1.0 (reference)			155 (136.3-173.6)	0.0 (reference)	
**25-70%**	253 (52.3)	84.4 (79.8-89.3)	0.0010	0.47 (0.29-0.76)	0.0021		187.4 (177.1-197.6)	32.4 (11.2-53.7)	0.0028
**70-100%**	107 (22.1)	88.9 (82.6-95.7)	0.0007	0.33 (0.17-0.65)	0.0015		197.8 (184.1-211.6)	42.9 (19.7-66)	0.0003

Disease free survival (DFS)	No. of patients (%)	Rate at 5 yr % (95% CI)	P value*	Unadjusted stratified by center<br>Hazard ratio (95% CI)	P value**	C-index (95% CI)	Restricted Mean Survival Time (RMST)<br>Months (95% CI)	Relative Months (95% CI)	P value***
**IS-2Level**						0.56 (0.52-0.6)			
**0-25%**	124 (25.6)	63.2 (55.1-72.4)		1.0 (reference)			146.9 (121.9-171.9)	0.0 (reference)	
**25-100%**	360 (74.4)	80.6 (76.5-84.9)	0.0006	0.59 (0.43-0.81)	0.0012		188.8 (172.5-205.1)	41.9 (12.1-71.8)	0.0059
**IS-3Level**						0.57 (0.52-0.62)			
**0-25%**	124 (25.6)	63.2 (55.1-72.4)		1.0 (reference)			141.4 (118-164.7)	0.0 (reference)	
**25-70%**	253 (52.3)	79.3 (74.3-84.6)	0.0018	0.61 (0.43-0.86)	0.0046		183.1 (164.9-201.2)	41.7 (12.1-71.3)	0.0058
**70-100%**	107 (22.1)	83.8 (76.8-91.5)	0.0053	0.55 (0.35-0.85)	0.0075		188.1 (160.7-215.4)	46.7 (10.8-82.6)	0.0109

STAGE II Very High Risk, T4 and VELIPI+ (Cohorts 1+2)

Time to recurrence (TTR)	No. of patients (%)	Rate at 5 yr % (95% CI)	P value*	Unadjusted stratified by center<br>Hazard ratio (95% CI)	P value**	C-index (95% CI)	Restricted Mean Survival Time (RMST)<br>Months (95% CI)	Relative Months (95% CI)	P value***
**IS-2Level**						0.6 (0.36-0.84)			
**0-25%**	29 (39.2)	48.9 (33.2-72)		1.0 (reference)			81 (50.4-111.7)	0.0 (reference)	
**25-100%**	45 (60.8)	80.8 (69.6-93.7)	0.0037	0.45 (0.17-1.15)	0.0964		136.4 (116.4-156.3)	55.3 (18.8-91.9)	0.0030
**IS-3Level**						0.62 (0.35-0.89)			
**0-25%**	29 (39.2)	48.9 (33.2-72)		1.0 (reference)			81 (50.4-111.7)	0.0 (reference)	
**25-70%**	30 (40.5)	79.1 (65.5-95.5)	0.0177	0.53 (0.2-1.42)	0.2075		131.5 (106.3-156.8)	50.5 (10.8-90.2)	0.0127
**70-100%**	15 (20.3)	84.4 (66.6-100)	0.0311	0.23 (0.04-1.37)	0.1061		90.8 (74.1-107.4)	36 (12.2-59.9)	0.0031

Disease free survival (DFS)	No. of patients (%)	Rate at 5 yr % (95% CI)	P value*	Unadjusted stratified by center<br>Hazard ratio (95% CI)	P value**	C-index (95% CI)	Restricted Mean Survival Time (RMST)<br>Months (95% CI)	Relative Months (95% CI)	P value***
**IS-2Level**						0.6 (0.38-0.82)			
**0-25%**	29 (39.2)	44.3 (29.3-66.9)		1.0 (reference)			60.3 (35.6-85)	0.0 (reference)	
**25-100%**	45 (60.8)	74 (61.8-88.5)	0.0011	0.44 (0.19-1.02)	0.0555		117.3 (91.7-142.9)	57 (21.5-92.6)	0.0017
**IS-3Level**						0.61 (0.36-0.86)			
**0-25%**	29 (39.2)	44.3 (29.3-66.9)		1.0 (reference)			60.3 (35.6-85)	0.0 (reference)	
**25-70%**	30 (40.5)	76.4 (62.5-93.3)	0.0043	0.46 (0.19-1.13)	0.0917		117.1 (88-146.2)	56.8 (18.7-95)	0.0035
**70-100%**	15 (20.3)	69.6 (48.7-99.6)	0.0370	0.36 (0.09-1.47)	0.1560		82.2 (64-100.4)	34 (9.9-58.1)	0.0057

STAGE II Very High Risk IS-2Level vs. LowRisk, T4 and VELIPI+ (Cohorts 1+2)

Time to recurrence (TTR)	No. of patients (%)	Rate at 5 yr % (95% CI)	P value*	Unadjusted stratified by center<br>Hazard ratio (95% CI)	P value**	C-index (95% CI)	Restricted Mean Survival Time (RMST)<br>Months (95% CI)	Relative Months (95% CI)	P value***
**Very High Risk IS-2Level vs. Low Risk**						0.55 (0.52-0.57)			
**LowRisk**	1360 (94.8)	85.6 (83.6-87.7)		1.0 (reference)			81 (50.4-111.7)	0.0 (reference)	
**VeryHighRisk-0-25%**	29 (2.0)	48.9 (33.2-72)	<.0001	4.49 (2.54-7.93)	<.0001		91.9 (55.3-128.6)	-79.2 (-116--42.3)	<.0001
**VeryHighRisk-25-100%**	45 (3.1)	80.8 (69.6-93.7)	0.2008	2.1 (1.06-4.16)	0.0329		136.4 (116.4-156.3)	-11.3 (-31.6-8.9)	0.2721

Disease free survival (DFS)	No. of patients (%)	Rate at 5 yr % (95% CI)	P value*	Unadjusted stratified by center<br>Hazard ratio (95% CI)	P value**	C-index (95% CI)	Restricted Mean Survival Time (RMST)<br>Months (95% CI)	Relative Months (95% CI)	P value***
**Very High Risk IS-2Level vs. Low Risk**						0.52 (0.5-0.53)			
**LowRisk**	1360 (94.8)	73.8 (71.4-76.3)		1.0 (reference)			60.3 (35.6-85)	0.0 (reference)	
**VeryHighRisk-0-25%**	29 (2.0)	44.3 (29.3-66.9)	<.0001	2.77 (1.7-4.51)	<.0001		65.2 (35.9-94.4)	-58.3 (-87.9--28.7)	0.0001
**VeryHighRisk-25-100%**	45 (3.1)	74 (61.8-88.5)	0.6983	1.16 (0.66-2.03)	0.6022		117.3 (91.7-142.9)	3.7 (-22.1-29.6)	0.7769

STAGE II High Risk IS-2Level vs. LowRisk, T4 OR VELIPI+ OR Perforation OR TLN <=12 OR Poor Differentiation (Cohorts 1+2)

Time to recurrence (TTR)	No. of patients (%)	Rate at 5 yr % (95% CI)	P value*	Unadjusted stratified by center<br>Hazard ratio (95% CI)	P value**	C-index (95% CI)	Restricted Mean Survival Time (RMST)<br>Months (95% CI)	Relative Months (95% CI)	P value***
**High Risk IS-2Level vs. Low Risk**						0.61 (0.56-0.65)			
**LowRisk**	551 (38.4)	89.2 (86.5-92.1)		1.0 (reference)			161.9 (149.8-174)	0.0 (reference)	
**HighRisk-0-25%**	278 (19.4)	72.7 (67.2-78.6)	<.0001	3.09 (2.06-4.64)	<.0001		161.9 (149.8-174)	-36.2 (-49.8--22.6)	<.0001
**HighRisk-25-100%**	605 (42.2)	86.2 (83.2-89.3)	0.1734	1.52 (1.04-2.21)	0.0295		231.8 (223.9-239.7)	-6.9 (-17.9-4.1)	0.2184

Disease free survival (DFS)	No. of patients (%)	Rate at 5 yr % (95% CI)	P value*	Unadjusted stratified by center<br>Hazard ratio (95% CI)	P value**	C-index (95% CI)	Restricted Mean Survival Time (RMST)<br>Months (95% CI)	Relative Months (95% CI)	P value***
**High Risk IS-2Level vs. Low Risk**						0.58 (0.55-0.6)			
**LowRisk**	551 (38.4)	78.3 (74.7-82)		1.0 (reference)			108.2 (95.9-120.5)	0.0 (reference)	
**HighRisk-0-25%**	278 (19.4)	62 (56.3-68.2)	<.0001	2.14 (1.67-2.74)	0.0000		108.2 (95.9-120.5)	-23.6 (-39.5--7.7)	0.0037
**HighRisk-25-100%**	605 (42.2)	73.8 (70.2-77.6)	0.6969	1.31 (1.06-1.62)	0.0136		149.6 (137.5-161.8)	5.6 (-14.4-25.6)	0.5810

STAGE II, T4 and N0 (Cohorts 1+2)

Time to recurrence (TTR)	No. of patients (%)	Rate at 5 yr % (95% CI)	P value*	Unadjusted stratified by center<br>Hazard ratio (95% CI)	P value**	C-index (95% CI)	Restricted Mean Survival Time (RMST)<br>Months (95% CI)	Relative Months (95% CI)	P value***
**IS-2Level**						0.68 (0.6-0.75)			
**0-25%**	72 (34.6)	46.3 (35.1-61)		1.0 (reference)			96 (69.9-122.1)	0.0 (reference)	
**25-100%**	136 (65.4)	84.6 (78.3-91.5)	<.0001	0.21 (0.11-0.4)	<.0001		176.8 (162.5-191.2)	80.9 (51.1-110.6)	<.0001

Disease free survival (DFS)	No. of patients (%)	Rate at 5 yr % (95% CI)	P value*	Unadjusted stratified by center<br>Hazard ratio (95% CI)	P value**	C-index (95% CI)	Restricted Mean Survival Time (RMST)<br>Months (95% CI)	Relative Months (95% CI)	P value***
**IS-2Level**						0.64 (0.58-0.69)			
**0-25%**	72 (34.6)	38.5 (28.2-52.5)		1.0 (reference)			69.9 (48.2-91.5)	0.0 (reference)	
**25-100%**	136 (65.4)	70.5 (62.7-79.1)	<.0001	0.31 (0.19-0.49)	<.0001		130.2 (112.9-147.6)	60.4 (32.6-88.1)	<.0001

STAGE II, T4 and N0 and No Chemo Treatment (Cohorts 1+2)

Time to recurrence (TTR)	No. of patients (%)	Rate at 5 yr % (95% CI)	P value*	Unadjusted stratified by center<br>Hazard ratio (95% CI)	P value**	C-index (95% CI)	Restricted Mean Survival Time (RMST)<br>Months (95% CI)	Relative Months (95% CI)	P value***
**IS-2Level**						0.78 (0.71-0.84)			
**0-25%**	45 (34.1)	40.8 (26.9-61.7)		1.0 (reference)			78.9 (46-111.8)	0.0 (reference)	
**25-100%**	87 (65.9)	87.5 (80.1-95.5)	<.0001	0.12 (0.05-0.28)	<.0001		178.2 (159.8-196.6)	99.3 (61.6-136.9)	<.0001

Disease free survival (DFS)	No. of patients (%)	Rate at 5 yr % (95% CI)	P value*	Unadjusted stratified by center<br>Hazard ratio (95% CI)	P value**	C-index (95% CI)	Restricted Mean Survival Time (RMST)<br>Months (95% CI)	Relative Months (95% CI)	P value***
**IS-2Level**						0.67 (0.61-0.74)			
**0-25%**	45 (34.1)	29.1 (17.7-47.8)		1.0 (reference)			54.1 (30.6-77.7)	0.0 (reference)	
**25-100%**	87 (65.9)	68.4 (58.8-79.5)	<.0001	0.25 (0.15-0.44)	<.0001		115 (94.1-135.8)	60.8 (29.4-92.3)	0.0002

* Logrank P Value. ** Wald P Value stratified by participating center. *** Restricted Mean Survival Time (RMST) P value. MSS: proficient Mismatch repair (pMMR).

Table S4. Multivariable analysis (Cohorts 1+2)

STAGE I-II	TTR Model (142/1075)*			DFS Model (341/1075)*		
	Hazard Ratio (95% CI)	P-value[1]	C-Index (95% CI)	Hazard Ratio (95% CI)	P-value[1]	C-Index (95% CI)
**Multivariable Stratified Cox Model**			0.7 (0.65-0.74)			0.61 (0.57-0.64)
**Immunoscore, 3-level (CD3/CD8 CT/IM)**						
Int *vs* Lo	0.47 (0.32-0.68)	0.0001		0.6 (0.47-0.78)	0.0001	
Hi *vs* Lo	0.29 (0.17-0.5)	<.0001		0.48 (0.35-0.65)	<.0001	
**Gender**						
Female vs Male	1.02 (0.73-1.43)	0.8930		1.02 (0.82-1.27)	0.8525	
**T-stage**						
T3 vs T1-2	1.99 (1.18-3.36)	0.0098		1.62 (1.19-2.22)	0.0023	
T4 vs T1-2	5.26 (2.92-9.49)	<.0001		2.42 (1.64-3.57)	<.0001	
**MSI Status (Derived)**						
dMMR vs pMMR	0.45 (0.25-0.82)	0.0087		0.96 (0.73-1.28)	0.7994	
**Sidedness**						
Distal vs proximal	1.34 (0.95-1.89)	0.0930		1.05 (0.84-1.32)	0.6407	

* (Events/Total); 1Stratified covariate Wald p-value; Stratified by center; MSI: deficient Mismatch repair (dMMR), MSS: proficient Mismatch repair (pMMR).

STAGE II MSS	TTR Model (114/644)*			DFS Model (230/644)*		
	Hazard Ratio (95% CI)	P-value[1]	C-Index (95% CI)	Hazard Ratio (95% CI)	P-value[1]	C-Index (95% CI)
**Multivariable Stratified Cox Model**			0.6 (0.54-0.67)			0.57 (0.52-0.61)
**Immunoscore, 3-level (CD3/CD8 CT/IM)**						
Hi *vs* Lo	0.45 (0.31-0.67)	0.0001		0.55 (0.41-0.73)	<.0001	
**Gender**						
Female vs Male	0.89 (0.61-1.3)	0.5483		1.03 (0.79-1.33)	0.8476	
**T-stage**						
T4 vs T3	2.6 (1.7-3.95)	<.0001		1.74 (1.26-2.42)	0.0009	
**Sidedness**						
Distal vs proximal	1.29 (0.88-1.88)	0.1867		1.04 (0.8-1.36)	0.7478	

* (Events/Total); 1Stratified covariate Wald p-value; Stratified by center.

STAGE I MSS	TTR Model (15/205)*			DFS Model (44/205)*		
	Hazard Ratio (95% CI)	P-value[1]	C-Index (95% CI)	Hazard Ratio (95% CI)	P-value[1]	C-Index (95% CI)
**Multivariable Stratified Cox Model**			0.77 (0.65-0.9)			0.64 (0.55-0.74)
**Immunoscore, 3-level (CD3/CD8 CT/IM)**						
Int *vs* Lo	0.41 (0.13-1.29)	0.1261		0.75 (0.34-1.68)	0.4848	
Hi *vs* Lo	0.08 (0.01-0.72)	0.0245		0.4 (0.14-1.11)	0.0776	
**Gender**						
Female vs Male	1.58 (0.51-4.94)	0.4320		0.75 (0.4-1.42)	0.3747	
**T-stage**						
T2 vs T1	2.37 (0.51-10.99)	0.2698		1.77 (0.77-4.06)	0.1798	
**Sidedness**						
Distal vs proximal	1.56 (0.53-4.63)	0.4198		1.2 (0.64-2.26)	0.5673	

* (Events/Total); 1Stratified covariate Wald p-value; Stratified by center. MSS: proficient Mismatch repair (pMMR).

STAGE II, T4N0	TTR Model (24/94)*			DFS Model (41/94)*		
	Hazard Ratio (95% CI)	P-value[1]	C-Index (95% CI)	Hazard Ratio (95% CI)	P-value[1]	C-Index (95% CI)
**Multivariable Stratified Cox Model**			0.76 (0.64-0.88)			0.74 (0.65-0.83)
**Immunoscore, 3-level (CD3/CD8 CT/IM)**						
Hi *vs* Lo	0.15 (0.05-0.46)	0.0009		0.2 (0.08-0.5)	0.0006	
**Gender**						
Female vs Male	1.33 (0.53-3.3)	0.5441		1.5 (0.74-3.02)	0.2605	
**Sidedness**						
Distal vs proximal	1.32 (0.49-3.57)	0.5821		1.31 (0.58-2.96)	0.5192	
**MSI Status (Derived)**						
dMMR vs pMMR	0.42 (0.12-1.48)	0.1775		0.33 (0.13-0.88)	0.0266	
**Differentiation**						
Moderate vs well	0.49 (0.1-2.35)	0.3759		0.93 (0.31-2.73)	0.8915	
Poor vs well	0.53 (0.06-4.53)	0.5631		2.09 (0.47-9.22)	0.3293	
**Mucinous colloid type**						
Yes vs No	1 (0.35-2.85)	0.9995		0.96 (0.44-2.12)	0.9278	
**VELIPI+**						
Yes vs No	0.79 (0.25-2.46)	0.6870		0.82 (0.35-1.93)	0.6452	

* (Events/Total); 1Stratified covariate Wald p-value; Stratified by center. MSS: proficient Mismatch repair (pMMR).

Sect on & Topic	No Item	details	Checklist
**TITLE OR ABSTRACT**	1	Ident f cat on as a study of diagnost c accuracy using at least one measure of accuracy	Yes
**ABSTRACT**	2	Structured summary of study design, methods, results, and conclusions (for specif c guidance, see STARD for Abstracts)	Yes
**INTRODUCTION**	3	Scient f c and clinical background, including the intended use and clinical role of the index test	Yes (novel Immune classif cat on of colon cancer, def ne high and low risk Stage I/III and Stage II colon cancer)
	4	Study object ves and hypotheses	Yes
**METHODS**			
**Study design**	5	Whether data collect on was planned before the index test and reference standard were performed (prospect ve study) or af er (retrospect ve study)	Yes, Retrospect ve study using pre-def ned test and scoring method and cutof , on 3 independent datasets
**Part cipants**	6	Eligibility criteria	Yes, (Inclusions: Pat ent with colon cancer; Age > 18 years; Pat ent had surgery performed prior to 2010. (5 years follow-up is recommended) ; Therapeut c procedures (surgery, radiotherapy, chemotherapy, biotherapy, ...) and follow-up registered; T1, T2, T3, T4 tumors; All N stages; pM0 at the t me of diagnosis ; Adjuvant chemotherapy is allowed). (Exclusion: Neo-adjuvant treatment, rectum cancer)
	7	On what basis potent ally eligible part cipants were ident f ed (such as symptoms, results from previous tests, inclusion in registry)	Yes, (pat ents with eligibility criteria from part cipat ng centers)
	8	Where and when potent ally eligible part cipants were ident f ed (set ng, locat on and dates)	Yes, (from part cipat ng centers)
	9	Whether part cipants formed a consecut ve, random or convenience series	Yes (pat ents with eligibility criteria from part cipat ng centers, randomly selected with 5 years follow-up)
**Test methods**	10a	Index test, in suf cient detail to allow replicat on	Yes (mult ple reproducibility data and details fro replicat on)
	10b	Reference standard, in suf cient detail to allow replicat on	Yes (AJCC/UICC-TNM)
	11	Rat onale for choosing the reference standard (if alternat ves exist)	Yes, (meta-analysis for available evidence of the associat on of immune cell inf ltrates with prognosis in various types of cancers)
	12a	Def nit on of and rat onale for test posit vity cut-of s or result categories of the index test, dist nguishing pre-specif ed from exploratory	Yes, (Test method was pre-def ned, cutof were def ned in the Training set, and subsequently validated in the independent datasets)
	12b	Def nit on of and rat onale for test posit vity cut-of s or result categories of the reference standard, dist nguishing pre-specif ed from exploratory	Yes (AJCC/UICC-TNM)
	13a	Whether clinical informat on and reference standard results were available to the performers/readers of the index test	Yes, (clinical informat on, percent les methods and cutof of the test are given)
	13b	Whether clinical informat on and index test results were available to the assessors of the reference standard	Reference (AJCC/UICC-TNM); Test was performed blinded to clinical data and reference standard. Reference standard was done blinded to Test.
**Analysis**	14	Methods for est mat ng or comparing measures of diagnost c accuracy	Yes, (several methods, including Cox mult variate analyses with Logrank P-values, Wald P-values, Chi2 risk contribut on, and Harrel's c-index are reported
	15	How indeterminate index test or reference standard results were handled	Yes, (pat ents with indeterminate data were excluded from the study)
	16	How missing data on the index test and reference standard were handled	Yes, (pat ents with missing data were excluded from the study)
	17	Any analyses of variability in diagnost c accuracy, dist nguishing pre-specif ed from exploratory	Yes, (Test was pre-specif ed, test method was pre-def ned, cutof were def ned in the Training set, and subsequently validated in the independent datasets)
	18	Intended sample size and how it was determined	Yes (>600 pat ents in each dataset, and >1200 Stage II, based on HR/P-values from previous studies)
**RESULTS**			
**Part cipants**	19	Flow of part cipants, using a diagram	Yes (see supplementary material)
	20	Baseline demographic and clinical characterist cs of part cipants	Yes (Table S1)
	21a	Distribut on of severity of disease in those with the target condit on	Yes (see supplementary tables)
	21b	Distribut on of alternat ve diagnoses in those without the target condit on	na
	22	Time interval and any clinical intervent ons between index test and reference standard	Yes (Standard of care from real-life cancers in each center)
**Test results**	23	Cross tabulat on of the index test results (or their distribut on) by the results of the reference standard	na
	24	Est mates of diagnost c accuracy and their precision (such as 95% conf dence intervals)	Yes (see tables)
	25	Any adverse events from performing the index test or the reference standard	na
**DISCUSSION**	26	Study limitat ons, including sources of potent al bias, stat st cal uncertainty, and generalisability	Yes
	27	Implicat ons for pract ce, including the intended use and clinical role of the index test	Yes (novel Immune classif cat on of colon cancer, def ne high and low risk Stage I/III and Stage II colon cancer)
**OTHER INFORMATION**	28	Registrat on number and name of registry	na
	29	Where the full study protocol can be accessed	Yes
	30	Sources of funding and other support; role of funders	Yes

Table S5: STARD checklist
